# UAV multispectral sensing and data-driven modeling for precision onion yield prediction

**DOI:** 10.3389/fpls.2025.1696730

**Published:** 2026-02-06

**Authors:** Sagar M. Wayal, Shardul Parab, Anusha Raj, Kiran Khandagale, Sanket Bhegde, Mukund Dawale, Indira Bhangare, Mahesh Khaire, Yogesh Kadam, Zafar Shaikh, V. Karuppaiah, Pranjali Gedam, Bhushan Bibwe, Sanket J. More, Lakesh K. Sharma, Vijay Mahajan, Suresh J. Gawande

**Affiliations:** 1 ICAR-Directorate of Onion and Garlic Research, Pune, India; 2 TIH Foundation for IoT & IoE, Mumbai, India; 3 Soil, Water, and Ecosystem Sciences Department, University of Florida, IFAS, Gainesville, FL, United States

**Keywords:** crop modeling, machine learning, multi-spectral sensors, onion production, precision agriculture, remote sensing, vegetation indices, yield prediction

## Abstract

The integration of unmanned aerial vehicle (UAV)-assisted remote sensing with the Internet of Things (IoT) and Internet of Everything (IoE) offers a robust platform for optimizing precision agriculture by capturing spatiotemporal variability in crop growth. In this context, the present study aimed to predict the bulb yield of rainy-season onion crops across four staggered planting dates using UAV-based multispectral imagery. Canopy reflectance mosaics acquired at key growth stages, along with vegetation indices (VIs), viz. NDVI, NDRE, SAVI, LAI, NORM2, and GNDVI, were extracted for yield modeling. Yield prediction models at three onion growth stages were developed and assessed using five machine learning algorithms: linear regression (lm), random forest (rf), support vector machine with radial kernel (svmRadial), gradient boosting (gbm), and elastic net regression (glmnet), with model training and evaluation performed using 10-fold cross-validation. Among these, random forest consistently outperformed the other models at all growth stages, showing promising results at the bulb development stage, with a training R^2^ = 0.944, RMSE = 1.919 t ha^-1^, MAE = 1.523 t ha^−1^, and a validation R² = 0.755, RMSE = 3.824 t ha^−1^, and MAE = 3.11 t ha^−1^. The support vector machine also demonstrated strong generalization (training R² = 0.787; validation R^2^ = 0.716), highlighting its predictive capability. Year-wise evaluation revealed notable interannual variability in model performance, with models trained on data from 2024 outperforming those from 2023. Overall, these results demonstrate the efficacy of UAV-derived multispectral sensing, combined with machine learning, as an effective, scalable, and timely approach for reliable onion yield prediction and decision support in rainy-season onion crops under varying agronomic conditions.

## 1 Introduction

Onion (*Allium cepa* L.) is a critical staple crop worldwide, valued for its nutritional benefits, extended storability, and central role in culinary applications. India stands as the world’s largest producer, cultivated across an area of 1.7 mha and contributing 30.19 Mt to global onion production in 2022–23 (FAOSTAT, 2025). Owing to its geographical location and diverse agro-climatic conditions, onion in India is cultivated across three distinct seasons, namely: (1) rainy season (June to September, referred to as *Kharif* onion); (2) post-rainy season (September to December, also called late *Kharif* onion); and (3) winter season (November–December to March, also called *Rabi* onion). Among these seasons, rainy-season onion production faces significant agronomic and economic challenges. Although rainy-season onions account for only 20%–25% of India’s total onion output, they play a crucial role in price stabilization because of their timely arrival, which coincides with the depletion of stored bulbs from the previous post-rainy or winter season (Gadge and Lawande, 2012). Yet, the rainy-season crop is highly susceptible to biotic stresses, including damping-off, anthracnose, purple blotch, and Stemphylium blight, owing to excessive moisture and unpredictable weather patterns, which often result in severe yield losses and sharp market price fluctuations (Moka et al., 2021; Salunke et al., 2022). Accurately forecasting onion yield is therefore essential for proactive supply chain management, early warning of shortages, and effective policy response, irrespective of the growing season.

Traditional methods for estimating crop yield rely on manual field surveys and destructive sampling, which are accurate at small scales but labor-intensive and impractical for timely, large-scale yield assessments. In contrast, satellite-based remote sensing has extended monitoring capabilities to larger areas (Usha and Singh, 2013). However, large-scale monitoring suffers from limitations related to spatial resolution, cloud cover, and infrequent revisit intervals, which are particularly problematic during the rainy season. These constraints necessitate the development of advanced, high-resolution, and timely monitoring tools for robust onion yield prediction under real-world farming conditions (Rahimi and Jung, 2024).

Recent advances in agricultural research have increasingly focused on deploying unmanned aerial vehicles (UAVs) for disease detection, plant health monitoring, and precise pesticide application due to their operational flexibility and close-range, high-resolution imaging (Faical et al., 2014). UAVs have become increasingly popular in precision agriculture because of their ability to capture high-resolution imagery at close range, surpassing many of the limitations of conventional satellite systems (Maes and Steppe, 2019). UAV-assisted multispectral imaging is now widely recognized for its value in modeling biotic and abiotic stresses, with numerous studies demonstrating successful automated disease detection in major crops, including wheat, potato, banana, cotton, peanut, and tomato (Su et al., 2018; Rodriguez et al., 2021; Ye et al., 2020; Wang et al., 2023; Chen et al., 2020; Abdulridha et al., 2020). These findings underscore the growing potential of UAV-based remote sensing for timely, data-driven crop management strategies.

Additionally, UAV-aided imagery has emerged as a powerful tool for crop growth monitoring and biomass estimation through non-destructive approaches, offering high spatiotemporal resolution that enhances its utility in agricultural applications (Fu et al., 2014). UAV platforms equipped with RGB, hyperspectral, and multispectral sensors have been effectively deployed to assess crop health and predict yield (Zhang and Kovacs, 2012). Vegetation indices (VIs), such as NDVI, ratio vegetation index (RVI), and leaf area index (LAI), have been extensively used to monitor crop growth and forecast yield across various crops, including rice (Din et al., 2017; Liu et al., 2025), wheat (Fu et al., 2020; Zhu et al., 2024), corn (Geipel et al., 2014; Bao et al., 2024), barley (Bendig et al., 2015), sugarcane (Ruwanpathirana et al., 2024), and onion (Córcoles et al., 2013). Although extensive UAV-based research exists for major cereals, relatively few studies have focused on commercially important vegetable crops such as onion. However, recent efforts have demonstrated the potential of UAV-derived multispectral imagery in onion research, including biomass monitoring (Ballesteros et al., 2018), yield prediction (Kang et al., 2020), and growth pattern analysis (Duarte-Correa et al., 2023; Farooqui et al., 2024). These emerging studies highlight the promise of UAV technology for improved monitoring and decision-making in onion cultivation. Earlier studies have effectively leveraged multiple machine learning algorithms to predict crop yield based on vegetation indices. Random forest (rf) has been validated for maize yield prediction using a ranking approach with NDVI, NDRE, and GNDVI (Ramos et al., 2020), while support vector machine models have been applied to wheat, potato, tomato, banana, and maize (Ayalew and Lohani, 2023). Gradient boosting (gbm) has been used for yield prediction in cassava, maize, plantains, potatoes, rice, sorghum, soybean, sweet potato, wheat, and yam (Mahesh and Soundrapandiyan, 2024). Despite these advances, significant gaps remain in evaluating and comparing the suitability of various vegetation indices for onion yield prediction. Leaf area index, a key indicator of crop photosynthesis, is widely recognized as a critical predictor, alongside plant height and crop surface models (Verger et al., 2014; Bendig et al., 2015, 2014). For onions and similar vegetable crops, plant height alone often lacks predictive reliability because of complex canopy structure and variable biomass accumulation. Moreover, most previous onion studies have focused on a single sowing or planting date, leaving intra-seasonal VI dynamics associated with staggered planting insufficiently explored. Understanding these temporal variations across growth stages is vital for developing robust and generalizable yield prediction models. This study introduces a growth stage-specific framework leveraging UAV-derived multispectral vegetation indices collected from the vegetative to bulb development stages across four staggered rainy-season plantings. The objectives of the current study were (1) to systematically analyze relationships between multiple vegetation indices and onion yield and (2) to assess the temporal dynamics of vegetation indices under staggered planting conditions. We hypothesize that high-resolution, UAV-derived multispectral vegetation indices collected at key growth stages, analyzed temporally and integrated with machine learning models, can accurately and robustly predict onion yield across staggered planting dates.

## 2 Materials and methods

### 2.1 Location of study area

**Figure 1:**
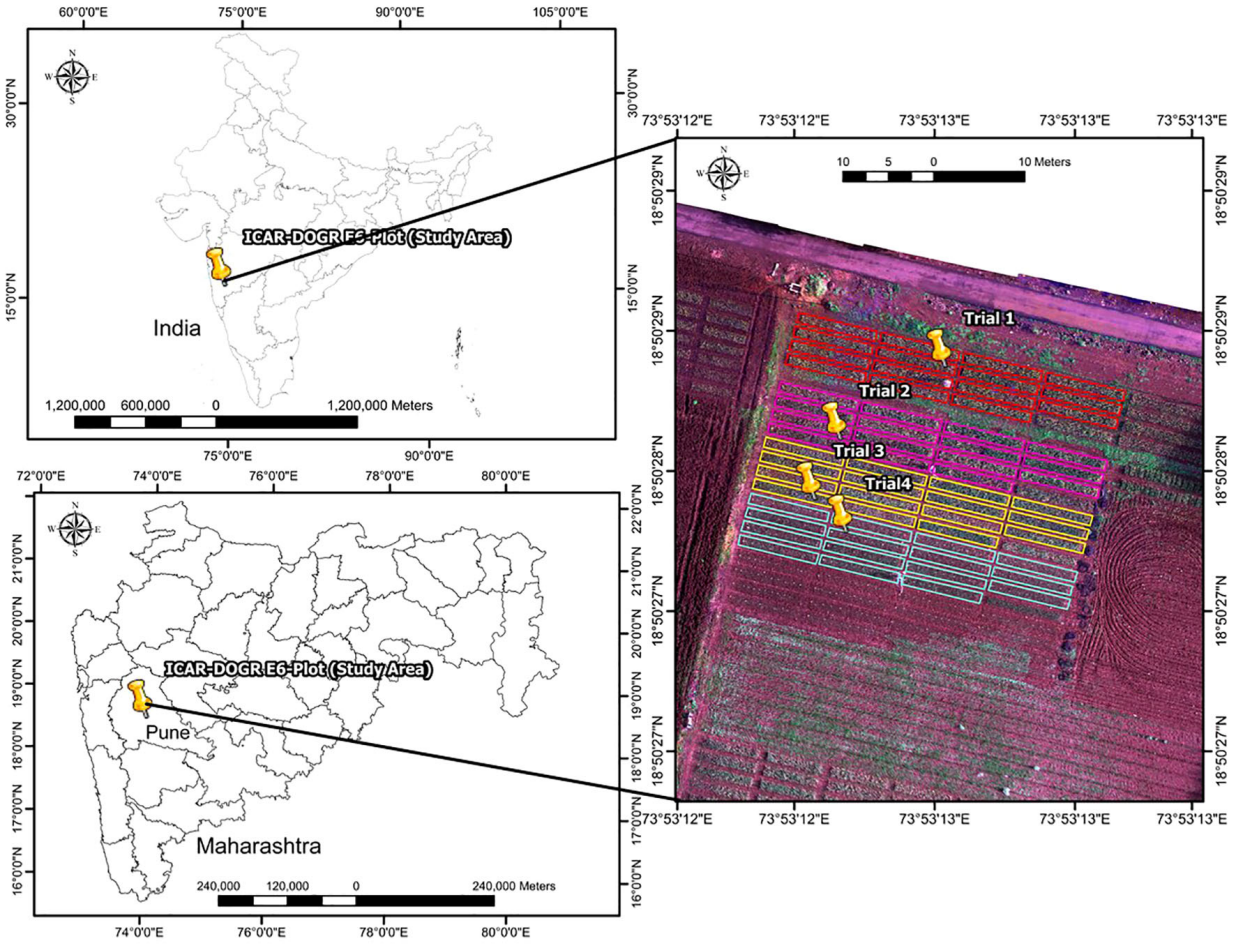
Map showing the geographical location of the study area used for UAV-based data collection and analysis.

**Figure 2:**
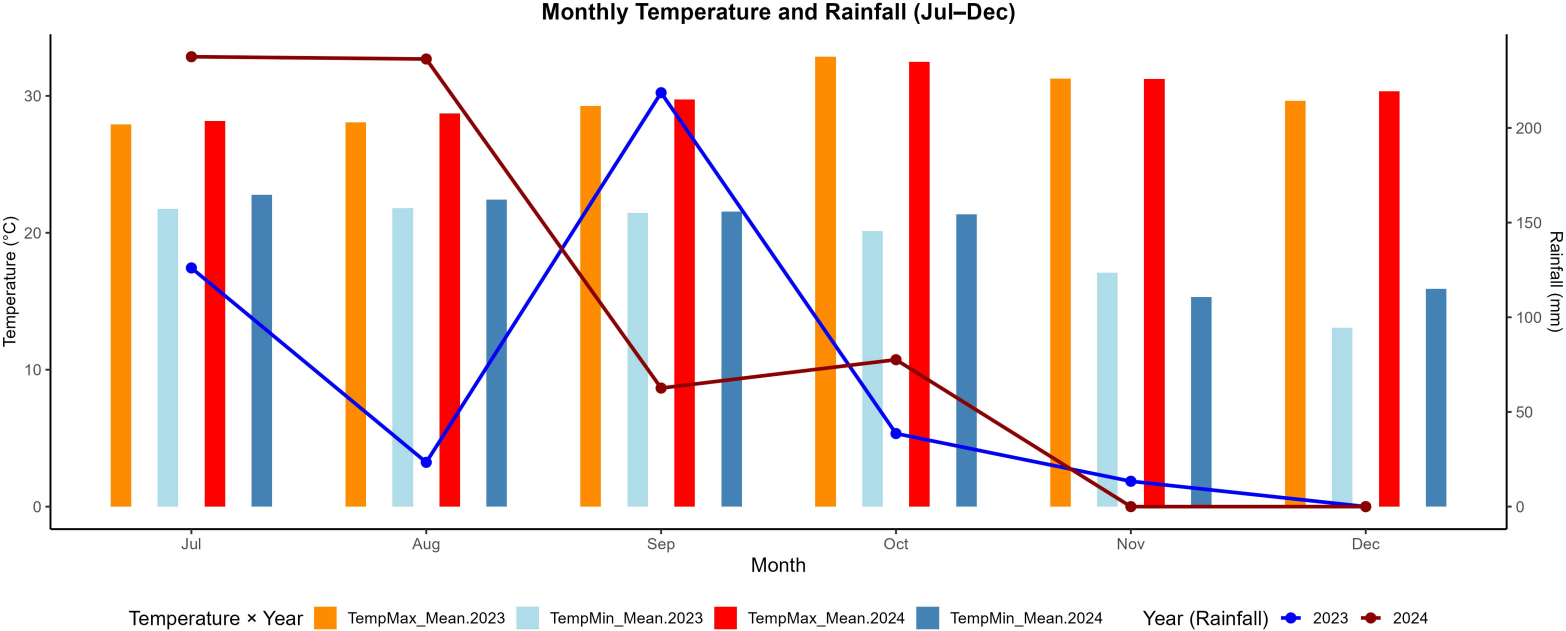
Temperature, relative humidity, average rainfall days, and precipitation of the study area during July-Decermber of 2023 and 2024 (source:IMD, Pune).

The experiment was conducted during the rainy season (July–December) in 2023 and 2024 at the ICAR–Directorate of Onion and Garlic Research, Pune, Maharashtra, India. The experimental site is geographically located at a latitude of 18°50’27.99” N and a longitude of 73°53’12.88” E (EPSG:4326; WGS 84/UTM zone 43N), as shown in **Figure 1**. **Figure 2** presents the average monthly maximum and minimum temperatures, as well as the total monthly precipitation recorded during the study period (i.e., July 1 to December 31) for both years. The study area lies in Maharashtra, India’s leading onion-producing state, contributing nearly 40% of the country’s total onion production (Sharma and Chauhan, 2024).

To capture intra-seasonal variability, four trials were established using staggered planting dates at 15-day intervals. Each trial followed a uniform experimental design comprising 15 subplots arranged in four rows. The first three rows comprised four subplots each, while the fourth row had three subplots. Each subplot measured 1 m × 8 m (8 m^2^) and was laid out on a broad bed furrow (BBF) system. A 1 m buffer was maintained between adjacent trials to minimize edge effects and ensure spatial independence during UAV flights and analysis. The nursery of the red onion cultivar ‘Bhima Super’, recommended for rainy-season cultivation, was raised on a BBF of 15 cm in height and 120 cm in top width, with a 45 cm furrow at 15-day intervals. Seedlings were transplanted 45–50 days after sowing at a spacing of 10 × 15 cm. Seedlings were dipped for at least 1 h in a solution containing carbendazim (50% WP; 1 g L^−1^) and carbosulfan (25% EC; 2 ml/L^−1^) before transplanting. The recommended fertilizer dose of 110:40:60:30 kg N:P:K:S ha^−1^ was applied, wherein the full dose of P and K, along with half of the N, was applied at transplanting, and the remaining N was top-dressed in two equal splits to ensure efficient nutrient utilization and sustained crop growth (Thangasamy and Lawande, 2015). The dates of transplanting (DOT) and dates of harvesting (DOH) are provided in **Table 1**. Onion bulbs were harvested at maturity when pseudostem lodging exceeded 50% (approximately 90–115 days after transplanting), and the marketable yield in each treatment was recorded. A total of 120 experimental plots were evaluated across four field trials conducted over two consecutive years (2023 and 2024).

**Table 1:** Details of multi-spectral data acquisition and intervals.

Trial	Year	DOT	DOH	UAV data collection date	Days after transplanting	Growth stage
1	2023	11.07.2023	05.10.2023	28.08.2023	48	Bulb Initiation
				14.09.2023	65	Bulb Development
	2024	03.08.2024	03.10.2024	29.08.2024	26	Vegetative
				20.09.2024	48	Bulb Initiation
				03.10.2024	61	Bulb Development
2	2023	24.07.2024	25.10.2023	28.08.2023	35	Vegetative
				07.09.2023	45	Bulb Initiation
				25.09.2023	63	Bulb Development
	2024	19.08.2024	04.11.2024	03.10.2024	45	Vegetative
				10.10.2024	52	Bulb Initiation
				30.10.2024	72	Bulb Development
3	2023	08.08.2023	02.12.2023	07.09.2023	37	Vegetative
				25.09.2023	48	Bulb Initiation
				20.10.2023	73	Bulb Development
	2024	03.09.2024	18.11.2024	10.10.2024	37	Vegetative
				30.10.2024	57	Bulb Initiation
				14.11.2024	72	Bulb Development
4	2023	25.08.2024	17.12.2024	25.09.2023	31	Vegetative
				20.10.2023	56	Bulb Initiation
				10.11.2023	77	Bulb Development
	2024	19.09.2024	04.12.2024	30.10.2024	41	Vegetative
				06.11.2024	48	Bulb Initiation
				22.11.2024	64	Bulb Development

### 2.2 Data collection and processing

#### 2.2.1 Acquisition of UAV images

A MicaSense RedEdge-P multispectral camera mounted on a UAV was employed to capture multispectral imagery across key crop growth stages. Flights were conducted under clear-sky conditions between 12:00 and 15:00 h at an altitude of 30 m above ground level and a flight speed of 2 m s^−1^. Image acquisition was planned with 80% forward overlap and 60% side overlap, resulting in 165–170 images per field per flight. The camera captured five multispectral bands (blue: 475 nm ± 32; green: 560 nm ± 24; red: 668 nm ± 16; red edge: 717 nm ± 12; near-infrared: 842 nm ± 46) and one high-resolution panchromatic band (634.5 nm ± 46) at each flight pass. The MicaSense RedEdge-P camera was equipped with a 6.3 mm diagonal sensor for multispectral imaging and an 11.1 mm sensor for panchromatic imaging, with a pixel size of 3.45 µm. The multispectral bands featured a resolution of 1450 x 1088 pixels (1.58 MP) with a 4:3 aspect ratio, while the panchromatic band had a resolution of 2464 × 2056 pixels (5.1 MP) and a 6:5 aspect ratio. The focal length was 5.5 mm for the multispectral bands and 10.3 mm for the panchromatic band, with a field of view of 49.6^0^ HFOV x 38.3^0^ VFOV (multispectral) and 44.5^0^ HFOV × 38.0° VFOV (panchromatic). The system captured 2–3 images per second, storing files in 16-bit TIFF raw format. Each image was georeferenced using the WGS 1984 datum and projected into UTM zone 43N to enable precise spatial analysis. Prior to each flight, radiometric calibration images were captured using standard reflectance panels placed on the ground, allowing accurate radiometric correction and reflectance conversion of UAV imagery. Detailed specifications of the camera system, flight parameters, and image acquisition schedule are summarized in **Tables 1**, **2**, respectively.

**Table 2:** Details of bands and bandwidth utilized for data collection.

Band number	Band name	Center	Bandwidth
1	Blue	475 nm	32 nm
2	Green	560 nm	24 nm
3	Red	668 nm	16 nm
4	Red edge	717 nm	12 nm
5	Near-infrared	842 nm	463 nm
6	Panchromatic	634.5 nm	463 nm

#### 2.2.2 Image processing

**Figure 3:**
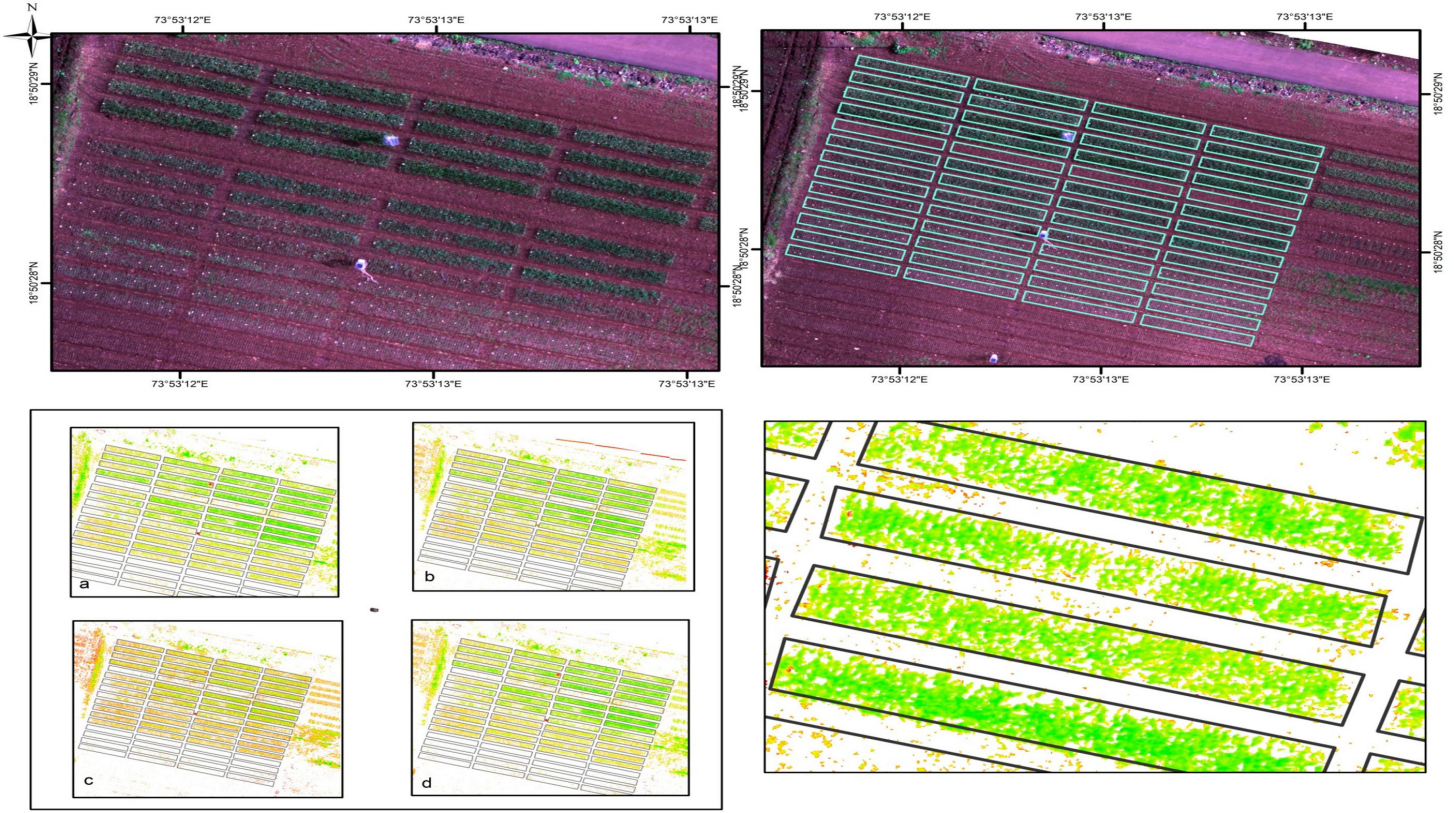
Orthomasaic map overlaid with field shapefiles and vegetation index layers, along with extracted zonal statistics for each plot.

**Figure 4:**
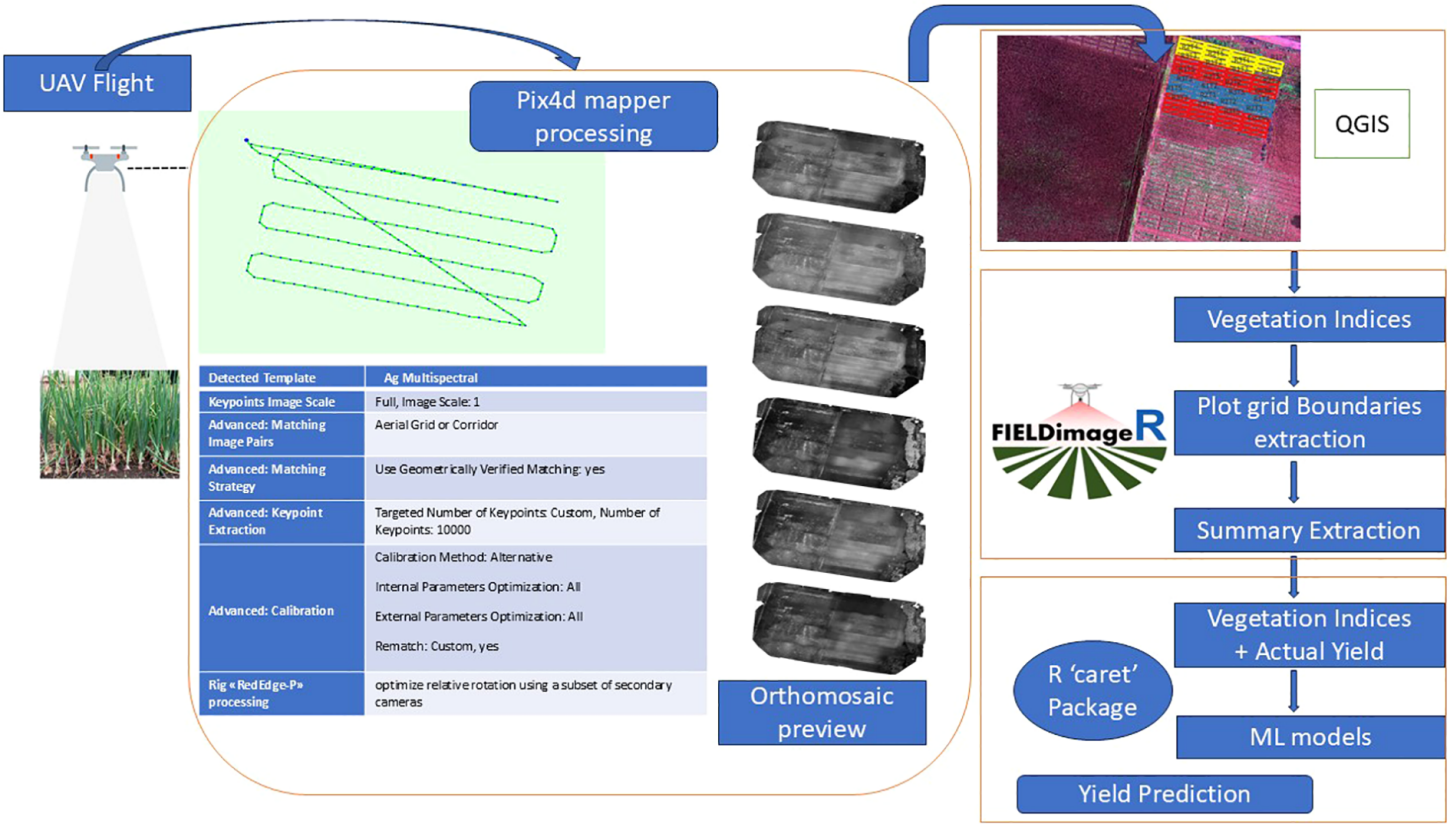
Workflow diagram illustrating the complete methodology adopted in this study, including UAV data acquisition, preprocessing, vegetation index generation, and subsequent analytical steps.

A set of images was processed using the automated processing template “Ag-Multispectral” in Pix4Dmapper 4.9.0 to perform cloud removal and radiometric correction, resulting in reflectance orthomosaics. Pre-processing included radiometric calibration, correction for sun sensor angles and irradiance, lens distortion correction (radial and tangential), and cloud removal. Radiometric corrections accounted for solar angle variations corresponding to the time of each flight (generally conducted between 12:00 and 15:00 h), ensuring consistent reflectance values across datasets. ArcGIS 10.3 was used to mosaic and composite the spectral bands. Onion field plots were delineated using shapefiles, and six multispectral bands were processed to remove soil pixels and eliminate edge artifacts from the orthomosaics. Vegetation index maps, illustrated in **Figures 3**, **4**, present zonal statistics extracted for each plot. Soil pixels were filtered using a mask based on the hue index, which classified each pixel as either soil or plant vegetation using a threshold value (Matias et al., 2020).

In this study, six vegetation indices were selected based on extensive literature evidence demonstrating their relevance to crop canopy structure, biomass accumulation, chlorophyll content, and vegetation vigor. These indices capture different aspects of plant physiology across growth stages, from early canopy development to maturity.

NDVI (normalized difference vegetation index) is one of the most widely used indicators of crop growth and canopy vigor. It is sensitive to chlorophyll content and canopy density, making it suitable for monitoring plant health and biomass, particularly during mid-season growth (Tucker, 1979). NDRE (normalized difference red edge index) provides greater sensitivity than NDVI during mid-to-late growth stages when canopies are dense, as the red-edge band avoids the saturation effect observed in the red band (Gitelson et al., 1996; Davidson et al., 2022). SAVI (soil-adjusted vegetation index) was designed to minimize the influence of soil background reflectance on vegetation signals, which is particularly important during early growth stages with sparse canopy cover (Huete, 1988). GNDVI (green normalized difference vegetation index) uses the green band instead of red, improving sensitivity to chlorophyll concentration and nitrogen status (Gitelson et al., 1996). NORM2 exploits the contrast between red and green reflectance and is sensitive to leaf pigmentation changes, plant stress, and senescence (Haboudance et al., 2004). LAI (leaf area index) is defined as the total leaf surface area per unit ground area. Empirical models using multispectral reflectance have been developed to estimate LAI (Haboudance et al., 2004). All vegetation indices were calculated from processed multispectral reflectance mosaics using the formulas provided in Equations 1–6 and **Table 3**. 

**Table 3:** Details of vegetation indices used, their formulas, and corresponding references.

Vegetation indices	Formula	Source
NDVI	$\frac{NIR-RED}{NIR+RED}$	Tucker (1979)
NDRE	$\frac{NIR-Red\;Edge}{NIR+Red\;Edge}$	Davidson et al. (2022), Eykerman (2022)
SAVI	$\frac{1.5*NIR-Red}{0.5+\left(NIR-Red\right)}$	Huete (1988)
GNDVI	$\frac{NIR-Green}{NIR+Green}$	Gitelson et al. (1996)
NORM2	$\frac{Red-Green}{Green+RED}$	Haboudane et al. (2004)
LAI	$\frac{3.618*2.5*\left(NIR-Red\right)}{\left(NIR+6*Red-7.5*Blue+1\right)}-\;0.118$	Haboudane et al. (2004)

(1) $$NDVI=\;\frac{NIR-RED}{NIR+RED}$$

(2) $$NDRE=\;\frac{NIR-Red\;Edge}{NIR+Red\;Edge}$$

(3) $$SAVI=\;\frac{1.5*NIR-Red}{0.5+\left(NIR-Red\right)}$$

(4) $$GNDVI=\;\frac{NIR-Green}{NIR+Green}$$

(5) $$NORM2=\;\frac{Red-Green}{Green+RED}$$

(6) $$LAI=\;\frac{3.618*2.5*\left(NIR-Red\right)}{\left(NIR+6*Red-7.5*Blue+1\right)}-\;0.118$$

Where: Red – Red band, Green – Green band, Blue – Blue band, NIR – Near infrared band, Red Edge – Red Edge band.

### 2.3 Statistical analysis

The linear relationship between each vegetation index (VI) and onion yield was assessed at the block level by performing Pearson’s correlation analysis. Additionally, principal component analysis (PCA) was applied to explore multivariate relationships and reduce dimensionality. Subsequently, multiple machine learning algorithms, including simple linear regression (lm), random forest (rf), support vector machine with radial kernel (svmRadial), gradient boosting machine (gbm), and elastic net regularized regression (glmnet), were evaluated separately for each growth stage, each year, and for the pooled dataset across all trials/planting dates to improve prediction accuracy. The selected algorithms represent a spectrum of modeling philosophies. Linear methods (lm, glmnet) provide interpretability and efficiency but may underperform with nonlinear relationships; ensemble learners (rf, gbm) improve robustness and handle complex interactions; and kernel-based approaches (svmRadial) capture nonlinear patterns but are sensitive to parameter tuning. Similar combinations have been widely adopted in crop yield prediction studies, where rf and svm consistently outperform classical regression models, and regularized models (glmnet) guard against overfitting (Jeong et al., 2016; Jiang et al., 2004; Hanuman et al., 2021). A k-fold cross-validation method was used to evaluate machine learning model performance to address the limited data generated by the experimental design. The dataset was randomly divided into k = 10 folds, and in each iteration, nine (10 − 1) folds (90% of the data) were used for training, while the remaining one fold (10%) was used for testing. A 10-fold cross-validation with a single repetition was employed to compute R^2^, RMSE, and MAE to assess the performance of the different ML models. This process was repeated until every fold had served as a validation set, ensuring that all observations contributed to both training and testing. Such resampling increases statistical efficiency, reduces bias, and provides more reliable error estimates than single-split approaches (Kohavi, 1995; Arlot and Celisse, 2010). Model hyperparameters were optimized via 10-fold cross-validation using caret’s default tuning grids. The final selected parameters were: rf (mtry = 2, using √p predictors per split), svmRadial (cost [C] = 0.25, σ = 0.86), gbm (50 trees, depth = 1, learning rate = 0.1), and glmnet (α = 0.1, λ = 0.058). This optimization strategy ensured robustness against overfitting and provided reliable estimates of performance generalization. All analyses were conducted in R v4.4.1 (R Core Team, 2024) using the caret package (Kuhn, 2008).

Principal component analysis is a multivariate statistical technique used to reduce the dimensionality of a dataset while preserving as much variability as possible. It does so by transforming the original variables into a new set of uncorrelated variables called principal components (PCs) (Paul et al., 2013). In accordance with PCA methodology, dimensionality was reduced by transforming the original matrix of vegetation indices into principal components. The decision to retain principal components was based on the Kaiser criterion (eigenvalues >1) and cumulative variance contribution (>95%), ensuring that the selected components explained most of the variability in the dataset while avoiding redundancy. The PCA transformation can be mathematically expressed as follows:

Given matrix $X\in \;{\mathbb{R}}^{n\times 7}$ of vegetation indices transformed data, PCA transformed X into $Z\in \;{\mathbb{R}}^{n\times 3}$ the transformation is defined as in Equation 7:

(7) Z= XW

Where: $X\in \;{\mathbb{R}}^{n\times 7}$ is the matrix of vegetation indices, n is no of samples, and p=7 vegetation indices. $Z\in \;{\mathbb{R}}^{n\times 3}$ is the matrix containing the first p=3 eigenvectors of the covariance matrix of X-scaled data. $W\in \;{\mathbb{R}}^{7\times 3}$ is the matrix containing the first 3 eigenvectors of the covariance matrix of X-scaled data.

Machine learning modeling

To assess the effectiveness of spectral indices in predicting onion yield across different growth stages, machine learning methods were trained using principal components (PCs) derived from vegetation indices (VIs). PCA was employed to reduce dimensionality while retaining 95% of the cumulative variance. To ensure unbiased model evaluation, PCA was fitted separately within each training fold during cross-validation, and the resulting transformation was applied to the corresponding test set. This procedure prevents data leakage from the test data into the training process. Five modeling algorithms—linear regression (lm), random forest (rf), support vector machine with radial kernel (svmRadial), gradient boosting machine (gbm), and elastic net regression (glmnet)—were trained using 10-fold cross-validation implemented through the caret package in R. All performance metrics presented in the Results represent cross-validated estimates derived from out-of-fold predictions. The dataset was partitioned into ten equal folds. In each iteration of the cross-validation process, nine folds were used for model training, including the application of PCA transformation, while the remaining one fold was held out as the validation set. This process was repeated ten times, ensuring that each fold served as the validation set once. Model performance was assessed using root mean square error (RMSE), coefficient of determination (R²), and mean absolute error (MAE). Final performance metrics were calculated by aggregating results across all 10 training and validation sets. A simple linear regression model based on vegetation indices was used for onion yield estimation (Equation 8) . The estimation accuracy of each vegetation index and planting date was evaluated, and the results were calculated as follows.

(8) $$Y\left(yield\right)=\beta_{1}x+\beta_{0}+\in$$

Where: Y is the Actual yield, and x is spectral indices like NDVI, NDRE, SAVI, GNDVI, LAI, and NORM2. $\beta_{1}$ is the intercept of the independent variable, and $\beta_{0}\;$ is constant.

Random forest regressor is an ensemble learning model that efficiently processes large-scale datasets by integrating multiple decision trees. This algorithm has the ability to obtain more accurate and stable predictions through noise reduction (Breiman, 2001). In this study, the random forest (rf) regressor was used to predict onion yield using principal components as independent variables, employing the rf method in R’s caret package with 10-fold cross-validation and the following parameters: $Z\in \;{\mathbb{R}}^{n\times 3}$ is the matrix of the principal components (explanatory variable), $y\in \;{\mathbb{R}}^{n}$ is the vector of onion yield values (dependent variable).

The random forest regressor consists of an ensemble of T decision trees, where T = n_estimator. Each decision tree t in the forest is trained on a bootstrap sample of the data and makes a prediction $h_{t}\left(Z\right)$. The final prediction $\hat{y}$ is obtained by averaging the predictions of all individual trees.

Mathematically, this is expressed in Equation 9.

(9) $$\hat{Y_{i}}=\frac{1}{T}\sum_{t=1}^{T}h_{t}\left(Z_{i}\right)$$

Where: $\hat{Y_{i}}\;$ is the predicted yield for the i^th^ sample, T is the number of trees (500 by default in rf), and $h_{t}\left(Z_{i}\right)$ is the prediction of i^th^ sample. Each tree grows with random subset of mtry = 2 features.

Support vector machine with a radial basis function (RBF) kernel is a robust model that creates an epsilon-insensitive tube around the regression function, which does not penalize predictions that fall within this tolerance band (Vapnik, 2013). The model is obtained by minimizing the total loss while maximizing the margin using the RBF (Gaussian) kernel function, with parameters epsilon (ϵ) = 0.1, regularization parameter (C) = 0.25, and sigma (σ) = 0.86. The mathematical formulation of the SVR model is presented in Equations 10-11. $Z\in \;{\mathbb{R}}^{n\times 3}$ is the matrix of the principal components (explanatory variable), $y\in \;{\mathbb{R}}^{n}$ is the vector of onion yield values (dependent variable).

The SVR function can be written as:

(10) f(Z)=ω.ϕ(ζ)+b

Where: $\omega \;\epsilon \;{\mathbb{R}}^{p}$ is the weight vector, ϕ(ζ) is the mapping function that transforms the input space Z into higher dimension feature space, and b is the bias term.

(11) $$\;min_{\omega ,\;b,\;\xi ,\;\xi^{*}\;}\;12{\left|\left|\omega \right|\right|}^{2}+C\sum_{i=1}^{n}\left(\xi_{i}+\xi_{i}^{*}\right)$$

Where ω,b  the model parameter, n is the number of sample points, and $\xi_{i},\xi_{i}^{*}>0\;$ is slack variables.

Gradient boosting is an ensemble supervised machine learning algorithm that combines multiple weak learners to create a final model (Friedman, 2001). The concept of boosting stems from the need to convert weak learners into stronger predictors. The gradient boosting algorithm requires a loss function to be optimized, a weak learner for making predictions, and an additive model for precise estimation. The mathematical formulation of the model follows a stage-wise procedure for each base learner from m = 1 to M iterations, as shown in Equation 12. Stage-wise additive model (Equation 12):

Stagewise additive model Equation 12:

(12) $$F_{m}\left(Z\right)=\;F_{m-1}\left(Z\right)+\;\nu \;h_{m}\left(Z\right)$$

Where, $h_{m\;}\left(Z\right)$ = weak learner, ν = 0.1 shrinkage learning rate from output.

Trees, build by minimizing loss $\sum_{i=1}^{n}L\;(y_{i},\;F_{m-1}\;\left(Z_{i}\right)+regularization$.

The glmnet regression model, as implemented in the caret package in R, is flexible for fitting generalized linear model functions of the independent variables. The model combines L1 (lasso) and L2 (ridge) regularization to balance feature selection and coefficient shrinkage (Friedman et al., 2010). The objective function of the elastic net model is shown in Equation 13.

Model formula: Equation 13. is the objective function of Elastic Net

(13) $$\underset{\beta_{0}\;\beta }{\min}\{\frac{1}{2N}\sum_{i=1}^{N}{\left(y_{i}-\beta_{0}-\sum_{j=1}^{p}Z_{ij}\beta_{j}\right)}^{2}+\lambda \left[\left(1-\alpha \right)\frac{∥\beta_{22}∥}{2}+∥\beta {∥}_{1}\right]\}$$

Where: y_i_ observed yield for i^th^ sample, Z_ij_ j^th^ principle component of i^th^ sample, $\beta_{0}\;and\;\beta$ intercept and regression coefficients, λ is penalty strentgth (tuned via cross validation) and α is mixing parameter.

### 2.4 Evaluation metrics

Multispectral and morphological data obtained from the experiment were used to establish rainy-season onion yield estimation models. Three evaluation metrics were used to assess model performance: coefficient of determination (R^2^) (Equation 14), root mean square error (RMSE) (Equation 15), and mean absolute error (MAE) (Equation 16). The coefficient of determination (R^2^) measures the proportion of variance explained by the regression model, while MAE and RMSE measure the average absolute error and the average magnitude of errors between predicted and observed values, respectively. $\bar{Y}$

(14) $$R^{2}=1-\frac{\sum_{1}^{N}{\left(Y_{i}-\hat{Y_{i}}\right)}^{2}}{\sum_{1}^{N}{\left(Y_{i}-\bar{Y}\right)}^{2}}$$

(15) $$RMSE=\sqrt{\frac{1}{N}\sum_{1}^{N}{\left(Y_{i}-\hat{Y_{i}}\right)}^{2}}$$

(16) $$MAE=\frac{\sum_{1}^{N}\left|Y_{i}-\hat{Y_{i}}\right|}{N}$$

Where Y_i_ and $\hat{Y_{i}}$ are the actual and predicted onion yield and $\bar{Y}$ is the mean value of yield, with N representing the sample size. A combination of software and statistical tools was used for image processing, data analysis, and predictive model fitting, including Pix4Dmapper, ArcGIS, and R, as illustrated in the workflow flowchart.

## 3 Results

The yield distribution across trials and years (**Supplementary Figure S1**) revealed substantial variation, with consistently higher yields recorded in the 2024 season compared with 2023. This improvement may reflect more favorable environmental conditions during 2024. Notably, Trials 3 and 4 in 2024 showed higher median yields and reduced variability, indicating more stable performance under those conditions. Complementing these findings, density plots of vegetation indices by year and crop growth stage (**Supplementary Figure S2**) highlighted clear spectral differences across stages and seasons. During the bulb development stage, higher densities of VIs such as NDVI, LAI, and SAVI were observed in 2024, suggesting improved canopy vigor and overall crop health compared with 2023.

### 3.1 Correlation of bulb yield and spectral indices

Vegetation indices exhibited varying degrees of correlation with final yield across different trials, growth stages, and years (**Supplementary Figures S3–S6**). Overall, NDRE, NDVI, and GNDVI consistently showed strong positive correlations, particularly during the bulb development stage across all four trials. In Trial 1, NDRE (R = 0.92, p < 0.001) and NDVI (R = 0.88, p < 0.001) were the strongest predictors during bulb development, while at earlier stages, such as bulb initiation and the vegetative phase, only moderate correlations were observed. In Trial 2, NDRE, GNDVI, and LAI in the 2024 season exhibited exceptionally strong correlations (R > 0.9) across all growth stages, confirming the reliability of these indices under favorable conditions. In contrast, the 2023 season showed lower and more inconsistent correlations compared with 2024, particularly during the vegetative and bulb initiation stages. Trial 3 demonstrated strong and stable performance of NDRE (R = 0.96, p < 0.001) and LAI (R = 0.92, p < 0.001) during the bulb development stage, along with moderate to strong associations at bulb initiation in 2024. The vegetative stage in Trial 3 showed moderate correlations, with GNDVI and NDVI performing slightly better than other indices. Trial 4 showed comparable trends, with NDRE and NORM2 emerging as the most consistent vegetation indices across stages, particularly in 2023. However, correlations during the vegetative stage in 2024 were generally weaker, with some indices, such as LAI and SAVI, exhibiting non-significant relationships. Across all trials, the bulb development stage consistently demonstrated the strongest correlations between vegetation indices and yield, reaffirming its role as the most appropriate growth stage for yield prediction. NDRE and NDVI emerged as the most robust and generalizable indices, followed by GNDVI and LAI. These results underscore the importance of stage-specific and trial-specific vegetation index selection to enhance modeling accuracy in precision agriculture.

### 3.2 Principal component analysis

**Figure 5:**
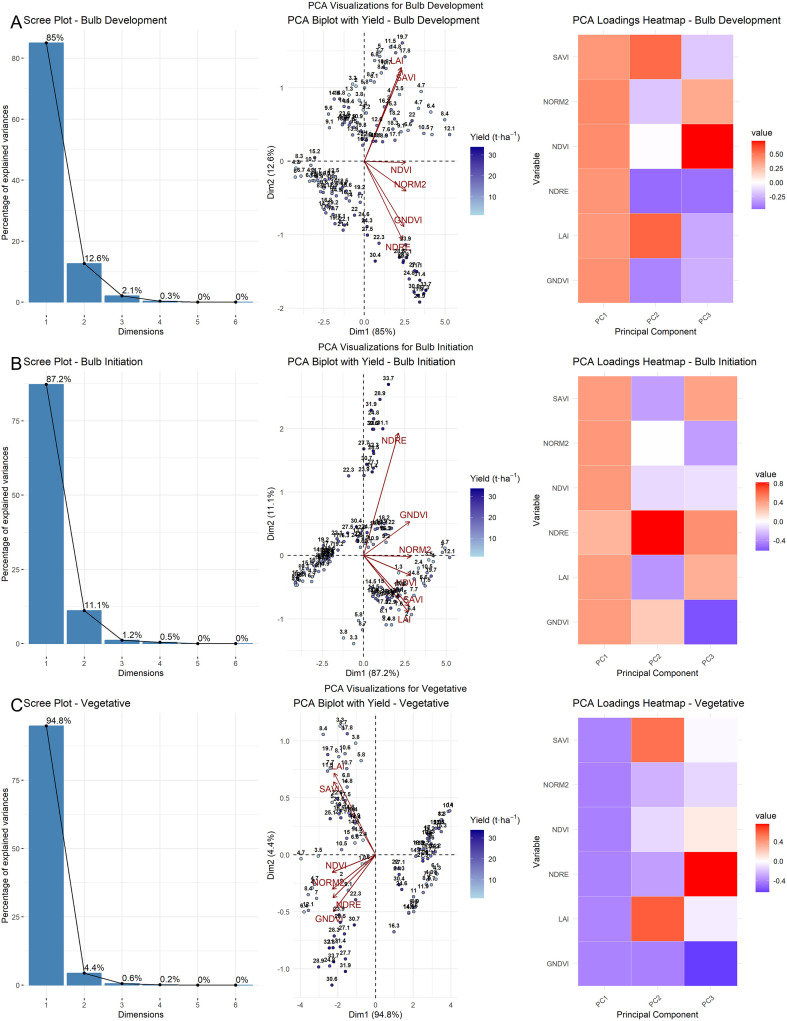
PCA of VIs for pooled-year data across crop growth stages: scree plot, biplot, and loading matrix heatmap. **(C)** Vegetative stage, **(B)** Bulb initiation stage, and **(A)** Bulb development stage.

Principal component analysis (PCA) reduced the complexity of the data across growth stages by analyzing spectral indices both separately and in pooled datasets for both years. For each growth stage, six vegetation indices (NDVI, NDRE, GNDVI, NORM2, SAVI, and LAI) were standardized and subjected to PCA; the variance contributions of the first (PC1), second (PC2), and third (PC3) principal components are presented on the X- and Y-axes (**Figure 5**). The loading matrices derived from PCA indicated the relative contributions of each original variable to the principal components. Overall, PC1 consistently captured the majority of the variance with substantial loadings from all VIs, indicating their strong collective influence. In 2023, the first two principal components (PC1 and PC2) accounted for most of the total variance across all growth stages, explaining 85.2% and 12.4% at the vegetative stage, 84.5% and 12.8% at bulb initiation, and 90.0% and 6.1% at the bulb development stage, respectively. In contrast, during the 2024 season, PC1 alone explained a dominant portion of the variance, capturing 98.4% at the vegetative stage, 96.0% at the bulb initiation stage, and 98.1% at the bulb development stage. This indicates a high degree of correlation among the indices, suggesting that they responded similarly during that season. For the pooled dataset, the first two components (PC1 and PC2) collectively explained most of the variance across all growth stages. Specifically, at the vegetative stage, PC1 and PC2 accounted for 94.1% and 4.4% of the variance, respectively; at the bulb initiation stage, 87.2% and 11.1%; and at the bulb development stage, 84.3% and 13.9%.

**Figure 6:**
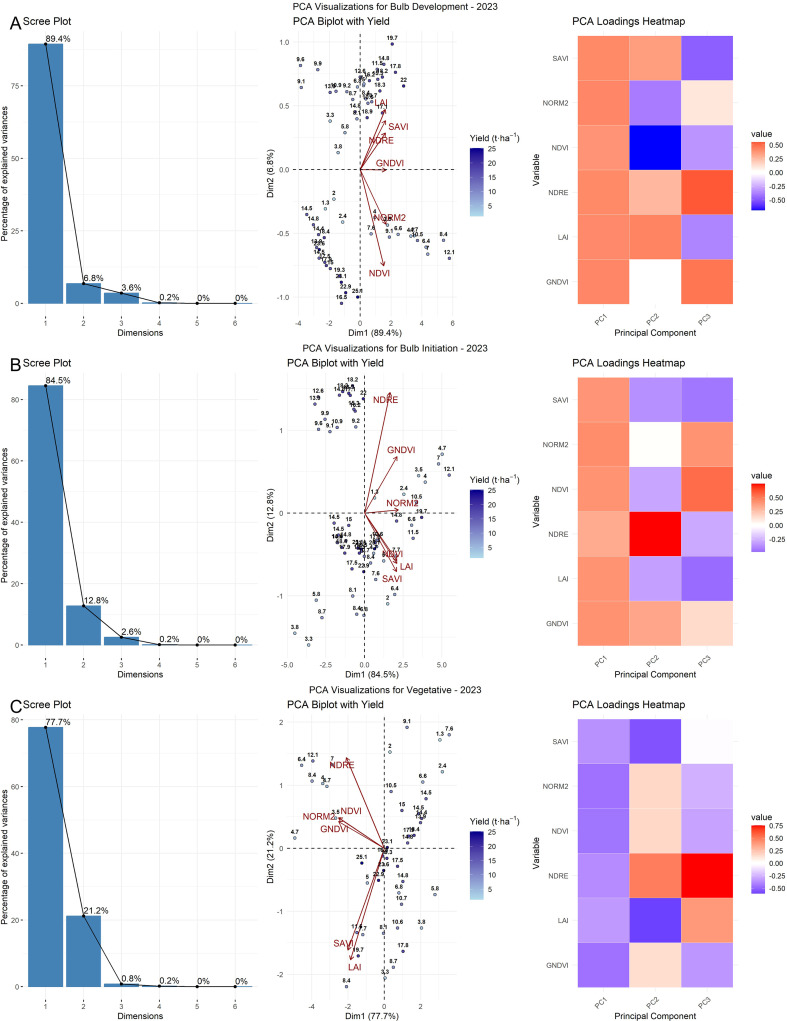
PCA of VIs for the year 2024 across crop growth stages: scree plot, biplot, and loading matrix heatmap. **(C)** Vegetative stage, **(B)** Bulb initiation stage, and **(A)** Bulb development stage.

At the vegetative stage in 2023, NDVI, NDRE, and NORM2 showed strong associations with PC1, while LAI and SAVI exhibited stronger associations with PC2. This pattern was confirmed by the corresponding loading matrix, indicating that LAI and SAVI made substantial contributions to PC2. In contrast, the 2024 biplot revealed tight clustering of all indices with high loadings on PC1, reflecting a high degree of correlation and a consistent physiological response. The loading matrix supported this observation, showing uniformly positive and high loadings for all indices on PC1, with minimal loadings on PC2, indicating coherence across indices (**Figure 6**).

The biplot of the pooled dataset exhibited intermediate behavior. While most indices aligned closely with PC1, similar to the pattern observed in 2024, LAI and SAVI showed slight deviations toward PC2, reflecting minor interannual variation. The pooled loading matrix reflected this pattern, showing strong contributions from all indices to PC1, with slight secondary loadings on PC2 for structure-related indices. At the bulb initiation stage, the 2023 PCA biplot showed a clear orientation of chlorophyll-related indices, such as NDVI, GNDVI, and NORM2, along PC1, indicating that these indices explained most of the variation during this stage. However, a moderate spread along PC2 was also observed. In contrast, the 2024 biplot revealed stronger coherence among vegetation indices. The pooled dataset biplot for the bulb initiation stage exhibited intermediate characteristics between the two years, with most indices, such as NDVI, NDRE, and NORM2, aligning strongly with PC1, while minor deviations were observed for certain indices along PC2. The pooled loading matrix showed strong contributions from key indices to PC1, with slight secondary loadings on PC2, especially for structure-related indices.

**Figure 7:**
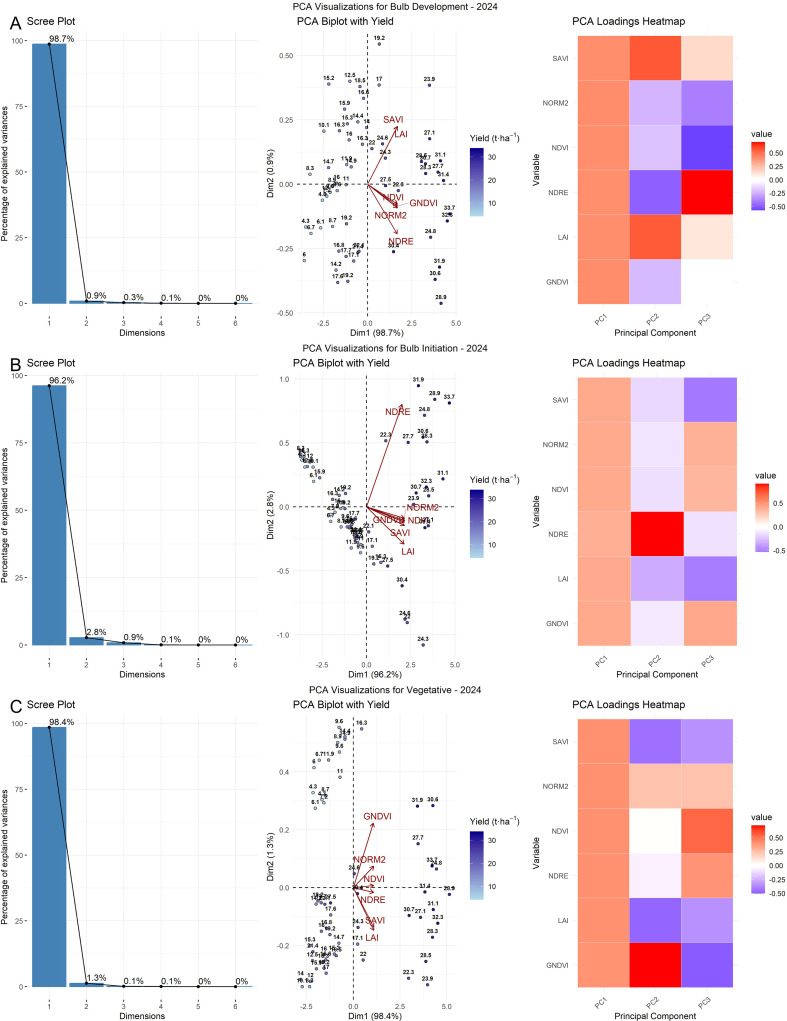
PCA of VIs for the year 2023 across crop growth stages: scree plot, biplot, and loading matrix heatmap. **(C)** Vegetative stage, **(B)** Bulb initiation stage, and **(A)** Bulb development stage.

At the bulb development stage, the 2023 PCA biplot revealed moderate separation of variables along the first two principal components. NDRE, GNDVI, and NORM2 were strongly aligned with PC1, while NDVI and LAI exhibited slight deviations toward PC2. The corresponding contribution heatmap showed strong positive loadings on PC1 and negative or mixed contributions on PC2 for certain indices (**Figure 7**). In contrast, the 2024 PCA biplot exhibited a more pronounced unidimensional pattern, with all indices—NDRE, GNDVI, NDVI, NORM2, and LAI—tightly clustered and positively aligned with PC1. The pooled PCA analysis again revealed intermediate characteristics. NDRE, GNDVI, and NORM2 maintained strong positive loadings on PC1, while SAVI and LAI diverged toward PC2, indicating slight interannual variation in canopy structural traits. The heatmap confirmed this pattern, with robust contributions from NDRE and GNDVI on PC1, while SAVI and LAI showed negative or mixed contributions on PC2. Importantly, PCA biplots overlaid with yield vectors indicated that yield was most closely associated with PC1, particularly in the direction of NDVI, NDRE, GNDVI, NORM2, and SAVI, implying that these indices are key predictors of bulb yield during the bulb development stage. This observation is further supported by the contribution heatmap, in which NDVI, NDRE, GNDVI, NORM2, and SAVI showed high positive contributions to PC1.

### 3.3 Machine learning tools

**Figure 8:**
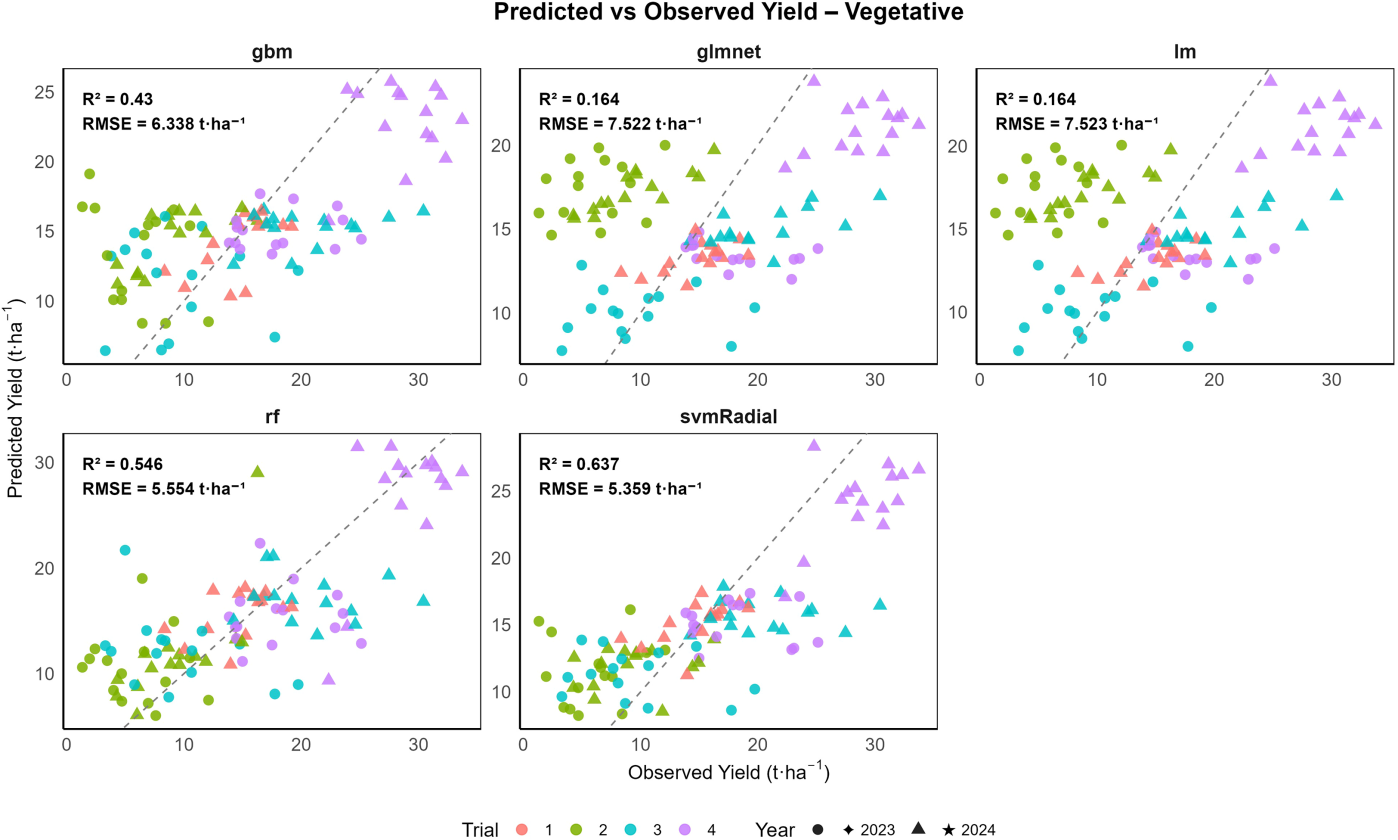
Validation performance of onion yield prediction models at the vegetative stage: observed versus predicted values using gbm, glmnet, lm, rf, and svmRadial with 10-fold cross-validation (n = 105).

**Figure 9:**
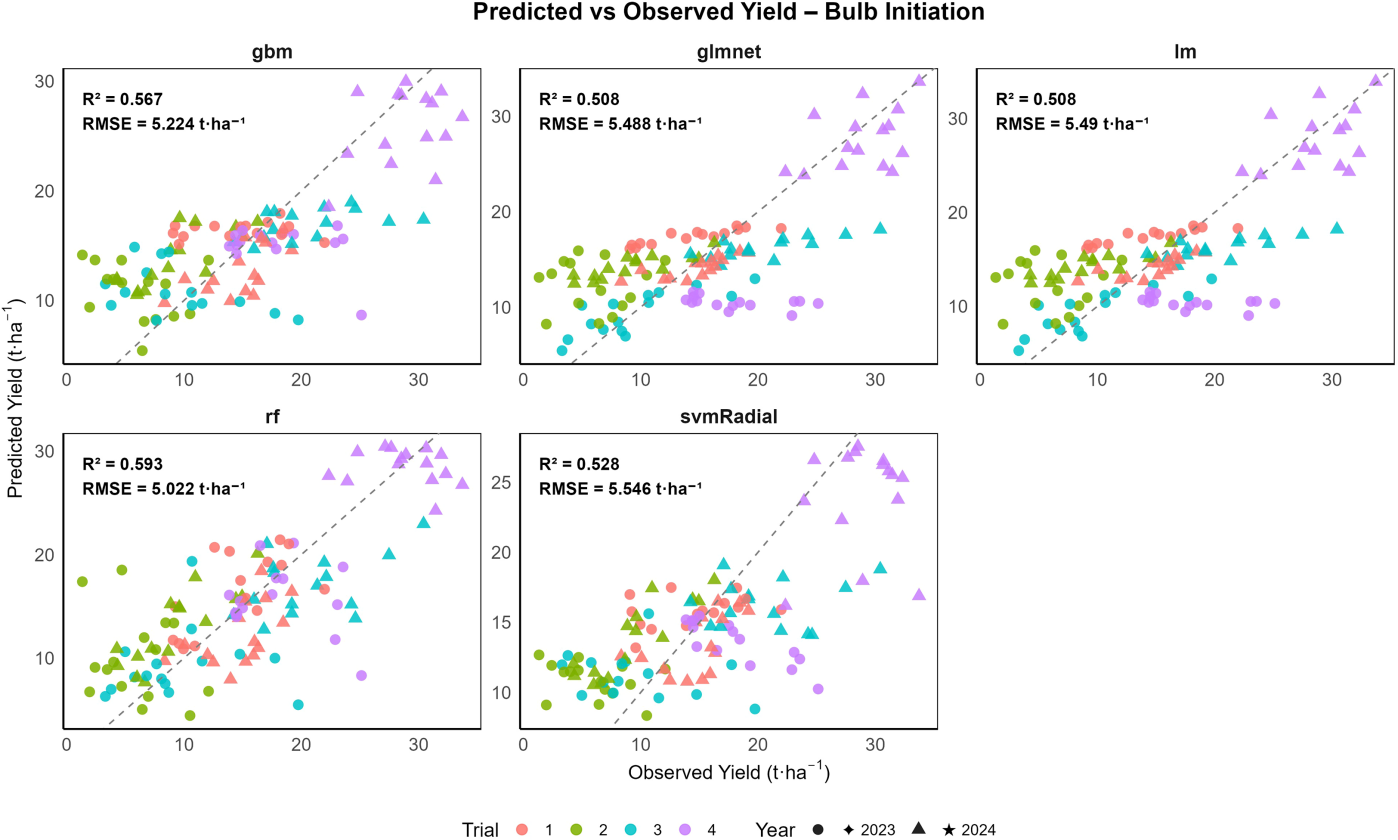
Validation performance of onion yield prediction models at the bulb initiation stage: observed versus predicted values using gbm, glmnet, lm, rf, and svmRadial with 10-fold cross-validation (n = 120).

**Figure 10:**
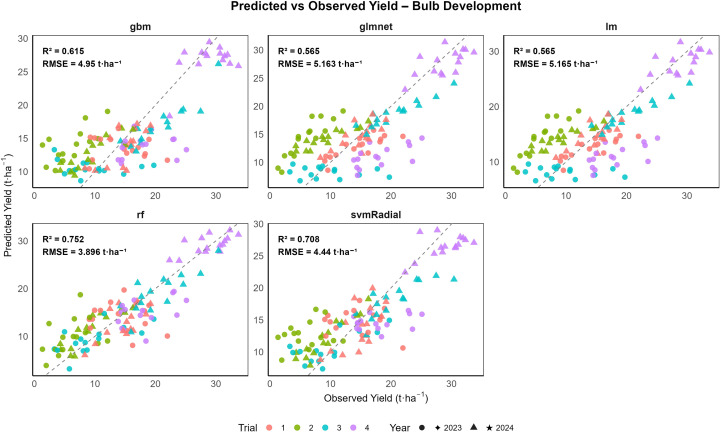
Validation performance of onion yield prediction models at the bulb development stage: observed versus predicted values using gbm, glmnet, lm, rf, and svmRadial with 10-fold cross-validation (n = 120).

The results are presented as boxplots of model performance metrics (RMSE and R^2^) for both combined and year-wise datasets (2023 and 2024) across different growth stages, as shown in **Supplementary Figures S7–S10**. These figures illustrate model performance across growth stages and demonstrate the stability and generalization ability of the machine learning (ML) models. To assess interannual variability, model performance was also evaluated separately for 2023 and 2024. For the combined dataset across both years and all growth stages, random forest (rf) consistently achieved the highest predictive accuracy, followed by svmRadial and gbm, while linear models (lm and glmnet) showed substantially lower performance (**Table 4**). Model performance at the vegetative stage is illustrated in **Figure 8**, where all five ML algorithms exhibited varying degrees of prediction accuracy. At the vegetative stage, the svmRadial model generalized well, with the lowest RMSE (4.891 t ha^−1^ for training and 5.276 t ha^-1^ for validation) and moderate R^2^ values (0.714 for training and 0.645 for validation), indicating moderate predictive ability. In contrast, linear regression and glmnet models exhibited higher errors (RMSE ≈ 7.39 t ha^-1^) and lower R^2^ values (0.193 for the training set and 0.234 for validation), indicating poor performance. The rf model exhibited the highest predictive power during training (R^2^ = 0.921), but its comparatively lower validation R^2^ of 0.579 indicated a tendency toward overfitting. A similar trend was observed at the bulb initiation stage (**Figure 9**), where rf achieved the highest R² (0.92) and the lowest RMSE (2.174 t ha^−1^) on the training set but showed reduced performance on the validation data (R^2^ = 0.654 and RMSE = 4.902 t ha^−1^). The gbm and svmRadial models followed with moderate generalization (gbm: RMSE = 4.362 t ha^−1^ for training and 5.119 t ha^−1^ for validation; R² = 0.71 for training and 0.63 for validation; svmRadial: RMSE = 4.849 t ha^−1^ for training and 5.572 t ha^−1^ for validation; R² = 0.67 for training and 0.58 for validation). During the bulb development stage, all ML models exhibited strong predictive capability (**Figure 10**). Among the evaluated models, rf demonstrated the highest overall accuracy, achieving the lowest RMSE of 1.919 ± 0.048 t ha^-1^ and the highest R^2^ of 0.944 ± 0.003 on the training set. Importantly, rf maintained moderate generalization, with a validation RMSE of 3.824 ± 0.787 t ha^-1^ and R^2^ of 0.755 ± 0.136. The svmRadial model also performed robustly, showing balanced metrics across training (R^2^ = 0.787 ± 0.026; RMSE = 3.912 ± 0.125 t ha^-1^) and validation (R² = 0.716 ± 0.158; RMSE = 4.388 ± 0.752 t ha^-1^), suggesting strong generalization ability. The gbm model showed moderate predictive performance, maintaining a training R^2^ of 0.743 ± 0.018 and a validation R^2^ of 0.649 ± 0.144, highlighting its potential as a robust predictive model. Linear models, including glmnet and lm, were comparatively less accurate but still demonstrated acceptable cross-validated performance, with R² values of 0.575 ± 0.208 and RMSE around 5.113 ± 0.912 t ha^-1^.

**Table 4:** Growth stage-wise evaluation of ML models for onion yield prediction using PCA-based VI features: combined data.

Growth_Stage	Model	Train	Test	Train	Test	Train	Test
		Mean_RMSE ± SD_RMSE		Mean_R^2^ ± SD_R^2^		Mean_MAE ± SD_MAE	
Vegetative	Gradient Boosting Machine (gbm)	5.502 ± 0.117	6.238 ± 0.987	0.618 ± 0.018	0.451 ± 0.174	4.322 ± 0.087	5.029 ± 0.926
Vegetative	Elastic net regression model (glmnet)	7.385 ± 0.130	7.408 ± 1.201	0.193 ± 0.027	0.234 ± 0.239	6.145 ± 0.137	6.215 ± 1.243
Vegetative	Linear Regression (lm)	7.385 ± 0.130	7.409 ± 1.205	0.193 ± 0.027	0.234 ± 0.239	6.145 ± 0.137	6.215 ± 1.246
Vegetative	Random Forest (rf)	2.507 ± 0.098	5.380 ± 1.177	0.921 ± 0.006	0.579 ± 0.219	1.866 ± 0.064	4.191 ± 0.796
Vegetative	Support Vector Machine (svmRadial)	4.891 ± 0.420	5.276 ± 0.899	0.714 ± 0.064	0.645 ± 0.180	3.760 ± 0.396	4.273 ± 0.654
Bulb Initiation	Gradient Boosting Machine (gbm)	4.362 ± 0.119	5.119 ± 1.003	0.719 ± 0.019	0.636 ± 0.157	3.457 ± 0.094	4.056 ± 0.781
Bulb Initiation	Elastic net regression model (glmnet)	5.403 ± 0.100	5.401 ± 0.902	0.523 ± 0.016	0.553 ± 0.157	4.187 ± 0.077	4.232 ± 0.783
Bulb Initiation	Linear Regression (lm)	5.402 ± 0.100	5.404 ± 0.899	0.523 ± 0.016	0.552 ± 0.157	4.178 ± 0.077	4.226 ± 0.782
Bulb Initiation	Random Forest (rf)	2.174 ± 0.042	4.902 ± 1.056	0.930 ± 0.004	0.654 ± 0.137	1.662 ± 0.023	3.813 ± 0.669
Bulb Initiation	Support Vector Machine (svmRadial)	4.849 ± 0.121	5.472 ± 0.871	0.670 ± 0.026	0.588 ± 0.148	3.725 ± 0.102	4.382 ± 0.650
Bulb Development	Gradient Boosting Machine (gbm)	4.207 ± 0.110	4.870 ± 0.993	0.743 ± 0.018	0.649 ± 0.144	3.415 ± 0.104	4.032 ± 0.801
Bulb Development	Elastic net regression model (glmnet)	5.014 ± 0.094	5.114 ± 0.893	0.589 ± 0.024	0.575 ± 0.208	3.985 ± 0.102	4.143 ± 0.766
Bulb Development	Linear Regression (lm)	5.013 ± 0.094	5.113 ± 0.912	0.589 ± 0.024	0.575 ± 0.208	3.973 ± 0.105	4.134 ± 0.797
Bulb Development	Random Forest (rf)	1.919 ± 0.048	3.824 ± 0.787	0.944 ± 0.003	0.755 ± 0.136	1.523 ± 0.036	3.110 ± 0.561
Bulb Development	Support Vector Machine (svmRadial)	3.912 ± 0.125	4.388 ± 0.752	0.787 ± 0.026	0.716 ± 0.158	3.048 ± 0.105	3.582 ± 0.463

Year-wise model evaluations revealed marked interannual variability, with 2024 consistently outperforming 2023 across all growth stages and model types. At the vegetative stage in 2024 (**Table 5**), all models showed strong predictive capability, with rf achieving the lowest RMSE of 3.554 ± 0.689 t ha^-1^ and the highest R^2^ of 0.866 ± 0.044, followed closely by svmRadial (R^2^ = 0.902 ± 0.058; RMSE = 4.071 ± 0.899 t ha^-1^). Even linear models, such as lm and glmnet, demonstrated high predictive power (R² = 0.822 ± 0.101; RMSE ≈ 4.26 t ha^-1^), indicating good generalization under favorable seasonal conditions. Conversely, in 2023 (**Table 4**), the same models exhibited substantially lower performance. Although rf remained the top performer (R² = 0.564 ± 0.251; RMSE = 4.978 ± 1.958 t ha^−1^), its generalization ability was reduced compared with 2024. The svmRadial model yielded an R² of 0.548 ± 0.344 and an RMSE of 4.071 ± 2.102 t ha^−1^. At the bulb initiation stage in 2024, rf again delivered robust performance (R² = 0.798 ± 0.102; RMSE = 3.696 ± 0.791 t ha^−1^), while gbm (R^2^ = 0.792; RMSE = 3.693 t ha^−1^) and svmRadial (R^2^ = 0.796 ± 0.294) also demonstrated high predictive accuracy. Linear models (lm and glmnet) maintained strong R² values of approximately 0.795. In contrast, during 2023, only rf (R^2^ = 0.595 ± 0.322; RMSE = 3.976 t ha^−1^), gbm (R^2^ = 0.505 ± 0.300; RMSE = 4.623 t ha^−1^), and svmRadial (R^2^ = 0.548 ± 0.273; RMSE = 4.490 ± 1.347 t ha^−1^) showed moderate performance. The bulb development stage demonstrated the highest model performance across both years. In 2024, gbm achieved the most accurate predictions (R² = 0.909 ± 0.038; RMSE = 2.933 ± 0.336 t ha^−1^), followed by rf (R^2^ = 0.889 ± 0.087; RMSE = 2.747 ± 0.807 t ha^-1^). Notably, linear models also performed exceptionally, with both lm and glmnet reaching R^2^ = 0.882 ± 0.056 and RMSE ≈ 3.0 t ha^−1^. In 2023, rf and svmRadial retained relatively good performance (R^2^ = 0.622 and 0.631; RMSE = 3.889 and 4.266 t ha^-1^, respectively), whereas the performance of linear models declined markedly, with reduced R² values and increased RMSE, likely reflecting year-to-year variability.

**Table 5:** Growth stage-wise evaluation of ML models for onion yield prediction using PCA-based VI features: Year-2024 data.

Growth_Stage	Model	Train	Test	Train	Test	Train	Test
		Mean_RMSE ± SD_RMSE		Mean_R^2^ ± SD_R^2^		Mean_MAE ± SD_MAE	
Vegetative	Gradient Boosting Machine (gbm)	3.146 ± 0.117	4.293 ± 0.989	0.858 ± 0.011	0.803 ± 0.130	2.474 ± 0.119	3.451 ± 0.690
Vegetative	Elastic net regression model (glmnet)	4.156 ± 0.134	4.258 ± 1.182	0.736 ± 0.019	0.822 ± 0.101	3.255 ± 0.096	3.458 ± 0.965
Vegetative	Linear Regression (lm)	4.155 ± 0.134	4.261 ± 1.187	0.736 ± 0.019	0.822 ± 0.101	3.251 ± 0.095	3.453 ± 0.968
Vegetative	Random Forest (rf)	1.702 ± 0.049	3.554 ± 0.689	0.957 ± 0.003	0.866 ± 0.044	1.323 ± 0.042	2.820 ± 0.578
Vegetative	Support Vector Machine (svmRadial)	3.405 ± 0.290	4.071 ± 0.899	0.890 ± 0.011	0.902 ± 0.058	2.674 ± 0.233	3.352 ± 0.849
Bulb Initiation	Gradient Boosting Machine (gbm)	2.796 ± 0.127	3.693 ± 0.550	0.883 ± 0.012	0.792 ± 0.094	2.340 ± 0.119	3.201 ± 0.610
Bulb Initiation	Elastic net regression model (glmnet)	3.608 ± 0.094	3.663 ± 0.878	0.801 ± 0.012	0.795 ± 0.119	2.942 ± 0.096	3.086 ± 0.810
Bulb Initiation	Linear Regression (lm)	3.606 ± 0.094	3.660 ± 0.890	0.801 ± 0.012	0.795 ± 0.119	2.957 ± 0.095	3.098 ± 0.819
Bulb Initiation	Random Forest (rf)	1.879 ± 0.072	3.696 ± 0.791	0.948 ± 0.004	0.798 ± 0.102	1.544 ± 0.071	3.179 ± 0.808
Bulb Initiation	Support Vector Machine (svmRadial)	4.435 ± 0.451	4.924 ± 1.065	0.841 ± 0.015	0.796 ± 0.111	3.368 ± 0.350	3.934 ± 0.898
Bulb Development	Gradient Boosting Machine (gbm)	2.150 ± 0.069	2.933 ± 0.336	0.930 ± 0.004	0.909 ± 0.038	1.795 ± 0.056	2.529 ± 0.406
Bulb Development	Elastic net regression model (glmnet)	3.010 ± 0.062	3.006 ± 0.533	0.862 ± 0.006	0.882 ± 0.056	2.478 ± 0.066	2.520 ± 0.549
Bulb Development	Linear Regression (lm)	3.006 ± 0.062	3.010 ± 0.519	0.862 ± 0.006	0.882 ± 0.056	2.479 ± 0.069	2.528 ± 0.547
Bulb Development	Random Forest (rf)	1.304 ± 0.052	2.747 ± 0.807	0.975 ± 0.002	0.889 ± 0.087	1.075 ± 0.052	2.343 ± 0.867
Bulb Development	Support Vector Machine (svmRadial)	3.212 ± 0.396	3.982 ± 0.815	0.897 ± 0.012	0.855 ± 0.141	2.543 ± 0.201	3.294 ± 0.540

Across all growth stages, the random forest model consistently outperformed other algorithms in terms of R^2^, RMSE, and MAE. This trend is evident when jointly considering **Tables 4**–**6**, where rf achieved the best or near-best performance in each case. The consistency of rf’s superior results reinforces the robustness of this algorithm for yield prediction based on PCA-derived vegetation index features. The support vector machine with radial kernel also demonstrated strong predictive capability, showing slightly lower R^2^ values than rf but minimal differences between training and validation sets, indicating better generalization and reduced overfitting.

**Table 6:** Growth stage-wise evaluation of ML models for onion yield prediction using PCA-based VI features: Year-2023 data.

Growth_Stage	Model	Train	Test	Train	Test	Train	Test
		Mean_RMSE ± SD_RMSE		Mean_R^2^ ± SD_R^2^		Mean_MAE ± SD_MAE	
Vegetative	Gradient Boosting Machine (gbm)	4.031 ± 0.244	5.604 ± 2.450	0.668 ± 0.034	0.497 ± 0.246	3.140 ± 0.197	4.773 ± 2.199
Vegetative	Elastic net regression model (glmnet)	6.183 ± 0.164	6.547 ± 1.525	0.062 ± 0.022	0.174 ± 0.241	5.247 ± 0.179	5.717 ± 1.372
Vegetative	Linear Regression (lm)	6.183 ± 0.164	6.550 ± 1.526	0.062 ± 0.022	0.174 ± 0.241	5.246 ± 0.180	5.719 ± 1.373
Vegetative	Random Forest (rf)	2.407 ± 0.207	4.979 ± 1.958	0.883 ± 0.021	0.564 ± 0.251	1.911 ± 0.155	4.230 ± 1.805
Vegetative	Support Vector Machine (svmRadial)	4.649 ± 0.366	4.837 ± 2.102	0.539 ± 0.084	0.548 ± 0.344	3.497 ± 0.371	4.189 ± 1.769
Bulb Initiation	Gradient Boosting Machine (gbm)	3.886 ± 0.181	4.623 ± 1.522	0.646 ± 0.025	0.505 ± 0.300	2.950 ± 0.127	3.533 ± 0.911
Bulb Initiation	Elastic net regression model (glmnet)	5.717 ± 0.139	5.581 ± 1.197	0.102 ± 0.020	0.218 ± 0.219	4.707 ± 0.129	4.743 ± 0.881
Bulb Initiation	Linear Regression (lm)	5.717 ± 0.139	5.581 ± 1.200	0.102 ± 0.020	0.218 ± 0.219	4.706 ± 0.129	4.743 ± 0.884
Bulb Initiation	Random Forest (rf)	2.189 ± 0.111	3.976 ± 1.884	0.897 ± 0.009	0.595 ± 0.322	1.570 ± 0.071	3.112 ± 1.324
Bulb Initiation	Support Vector Machine (svmRadial)	4.371 ± 0.252	4.490 ± 1.347	0.557 ± 0.059	0.548 ± 0.273	3.113 ± 0.194	3.561 ± 0.818
Bulb Development	Gradient Boosting Machine (gbm)	3.793 ± 0.134	4.807 ± 1.555	0.757 ± 0.023	0.485 ± 0.283	3.004 ± 0.134	3.855 ± 1.078
Bulb Development	Elastic net regression model (glmnet)	5.936 ± 0.149	6.069 ± 1.348	0.032 ± 0.015	0.208 ± 0.244	4.933 ± 0.145	5.153 ± 1.173
Bulb Development	Linear Regression (lm)	5.936 ± 0.149	6.071 ± 1.350	0.032 ± 0.015	0.208 ± 0.244	4.932 ± 0.145	5.153 ± 1.175
Bulb Development	Random Forest (rf)	1.898 ± 0.128	3.889 ± 0.934	0.919 ± 0.007	0.622 ± 0.247	1.525 ± 0.078	3.249 ± 0.665
Bulb Development	Support Vector Machine (svmRadial)	3.943 ± 0.139	4.266 ± 1.449	0.665 ± 0.043	0.631 ± 0.254	2.966 ± 0.142	3.546 ± 1.010

## 4 Discussion

Timely and precise monitoring of crop growth and health is essential for optimizing agricultural management, improving yield forecasting, and supporting resource-efficient interventions (Cheng et al., 2016). In this context, UAV-assisted remote sensing has seen substantial advancements in agriculture over the past decade. UAVs provide the unique ability to collect high-resolution, real-time spatiotemporal data that reveal subtle changes in crop growth dynamics and canopy architecture (Calera et al., 2017). While these technologies have been extensively utilized for cereals and high-value horticultural crops (Zhou et al., 2017; Li et al., 2020), their application to onion—a shallow-rooted and leaf-specific crop with distinctive canopy and phenological traits—remains relatively novel. The present study investigated the utility of multispectral UAV imagery for predicting bulb yield in rainy-season onion cultivated under four different planting dates. Planting date is a critical determinant influencing onion growth and productivity because of its effects on thermal time accumulation, radiation interception, and photoperiod sensitivity (Brewster, 2008; Devulkar et al., 2015). The predictive performance of different vegetation indices (VIs), especially when measured at key growth stages, was assessed for yield estimation. Results indicated that remote sensing-based monitoring, when synchronized with critical phenophases, enables reliable prediction of final bulb yield across varying planting windows. These findings corroborate earlier reports by Pinter et al. (2003), Sakamoto et al. (2013), and Sun et al. (2020), which suggest that phenology-linked spectral sensing enhances the accuracy of crop modeling.

Present study demonstrated significant differences in vegetative vigor, bulb initiation timing, and canopy development across the four planting dates. These differences influenced spectral reflectance patterns and, consequently, yield prediction accuracy. In early-transplanted onions, the longer vegetative period led to denser canopies and increased chlorophyll accumulation, whereas late-transplanted onions experienced accelerated phenological progression due to reduced accumulation of growing degree days. Consequently, canopy reflectance and associated VIs exhibited varying levels of saturation and sensitivity across growth stages and planting dates. This dynamic interaction underscores the importance of timing UAV data acquisition to specific crop developmental phases, as emphasized by Tucker et al. (2005) and Yang et al. (2020).

Among the VIs evaluated, NDVI, NDRE, SAVI, LAI, NORM2, and GNDVI exhibited consistent relationships with final bulb yield, particularly when measured during the bulb development stage. NDVI, one of the most widely used indices, showed moderate to strong correlations with yield but tended to saturate under conditions of high LAI. This saturation effect, reported in several previous studies (Haboudane et al., 2004; Zarco-Tejada et al., 2016), limits NDVI’s effectiveness during late vegetative or pre-bulking phases when canopy closure approaches 100%. Conversely, indices such as GNDVI outperformed NDVI in dense canopies because of their greater sensitivity to subtle changes in chlorophyll content and structural biomass. GNDVI uses the green band instead of the red band, thereby reducing saturation effects and more effectively capturing nitrogen-induced variability in leaf pigment concentration (Delegido et al., 2011).

SAVI, which is designed to minimize the influence of soil background reflectance during early growth stages, was especially useful when canopy coverage was below 50%, as observed in early DAT imagery. The soil adjustment factor incorporated in this index enhances stability under partially vegetated conditions, consistent with findings by Mulla (2013) and Johansen et al. (2019). Notably, VI performance varied not only with crop growth stage but also across different planting dates. For example, NDVI exhibited relatively stable performance in early-planted onions, whereas GNDVI performed better in late-transplanted onions. This variation may be attributed to differences in canopy geometry, radiation use efficiency, and stress accumulation under varying environmental regimes. Sakamoto et al. (2013) and Xue and Su (2017) similarly reported that environmental and physiological shifts associated with planting time influence spectral responses and require tailored modeling strategies.

Canopy reflectance and its derived indices are fundamentally linked to chlorophyll content, leaf area index, and biomass accumulation, all of which are influenced by nutrient management, water availability, and plant stress status. The chlorophyll-rich leaves of actively growing onions strongly absorb red light and reflect near-infrared (NIR) radiation, forming the basis for vegetation indices such as NDVI and GNDVI. However, additional biophysical factors, including leaf angle distribution and canopy shadowing, can modulate these reflectance patterns. Such structural attributes often vary with cultivar, planting density, and growth rate, highlighting the need for location- and variety-specific calibration of yield prediction models (Xue and Su, 2017; Zhang et al., 2020).

Environmental factors such as solar radiation, relative humidity, temperature, and soil moisture played a pivotal role in the spectral performance of vegetation indices (VIs). These variables influence the physiological status of the crop, altering reflectance characteristics by affecting water content, pigment concentration, and stomatal conductance. For instance, cloud cover during image acquisition may lower near-infrared (NIR) reflectance because of reduced canopy temperature and transpiration rates. Sakamoto et al. (2013) and Tucker et al. (2005) emphasized the need to include environmental covariates in spectral models to reduce prediction error and improve model robustness.

The regression models developed in this study, based on both single and combined VIs, achieved moderate to high prediction accuracy (R² = 0.60–0.82). Models utilizing combined VIs, which integrate both pigment-sensitive and structural indices, consistently outperformed those built on individual indices, demonstrating that the synergistic use of complementary spectral information enhances yield estimation. These findings align with the results of Chlingaryan et al. (2018) and Liakos et al. (2018), who emphasized that multivariable modeling better captures the complex interactions underlying crop productivity.

Although linear regression models offer simplicity and ease of interpretation, their performance is constrained by assumptions of linearity and a limited capacity to handle noise and multicollinearity among predictor variables. Onion growth and yield, being influenced by numerous interacting physiological and environmental variables, may therefore benefit from more sophisticated modeling approaches. In this context, machine learning algorithms such as linear regression (lm), random forest (rf), support vector machine with radial kernel (svmRadial), gradient boosting machine (gbm), and elastic net regression (glmnet) have demonstrated superior performance in agricultural applications by capturing complex, nonlinear relationships (Liakos et al., 2018; Chlingaryan et al., 2018). Future research could leverage these advanced algorithms to enhance yield prediction accuracy and robustness, particularly under highly variable field conditions or in multi-environment trials.

Temporal dynamics emerged as a crucial factor influencing the strength of relationships between vegetation indices and yield. The study observed that spectral data acquired during the bulb development stage (approximately 60–70 DAT) exhibited the highest correlation with final bulb yield. This period coincides with peak leaf area, optimal radiation interception, and maximum canopy greenness (Brewster, 2008). At this phase, vegetative traits are closely linked with yield potential, enabling accurate prediction (Zhou et al., 2017; Zaman-Allah and Vergara, 2015; Junior et al., 2025). However, for early planting scenarios in which vegetative growth is prolonged, the optimal imaging window may shift slightly forward or backward depending on crop phenology and local microclimate conditions. Thus, stage-specific image acquisition strategies should be aligned with the onion crop’s physiological development.

Consistent with these findings, superior model performance was observed for the 2024 data compared with 2023, underscoring interannual yield variability and suggesting that volatile weather dynamics contributed to deviations in VI values and actual yield across both years (Huang et al., 2021; Knight et al., 2024). Accurate crop yield prediction is essential for agricultural planning and decision-making, enabling stakeholders to optimize resource allocation and mitigate food security risks (Clercq and Mahdi, 2024). Yield prediction remains challenging because of complex interactions between crop development and yield-affecting environmental variables such as weather and soil fertility status (Pham et al., 2022). Interannual yield variation—year-to-year fluctuation in crop yields—further complicates prediction efforts because of the dynamic interplay among environmental factors and management practices (Mohan et al., 2025). Mitigating interannual yield variation requires a multifaceted strategy combining genetic improvements, adaptive agronomic practices, and technology integration. Crop diversification can buffer against environmental stresses and pest outbreaks. Adaptive techniques such as laser land leveling, timely sowing, efficient use of beneficial microbes, and precision irrigation enhance climate resilience (Rakshit et al., 2022). The deployment of drought-tolerant varieties and optimized water use is especially vital in water-scarce regions. In addition, practices such as conservation tillage, crop rotation, and organic amendments improve soil health and enhance yield stability under variable conditions (Dhankher and Foyer, 2018; Ortuani et al., 2019).

Furthermore, the consistent performance of vegetation indices across growth stages and planting dates highlights their potential for developing generalized models for bulb yield prediction. However, model transferability is constrained by factors such as soil background reflectance, crop management practices, sensor characteristics, and atmospheric conditions (Hsiao et al., 2019; Khaki et al., 2020). These challenges necessitate rigorous image calibration, including the use of reflectance panels, ground control points (GCPs), and standardized processing algorithms to ensure data consistency across flights and growing seasons (Zarco-Tejada et al., 2001; Tucker et al., 2005). Planting time also plays a critical role in shaping onion crop physiology. Early planting in this study resulted in extended vegetative growth, greater biomass accumulation, and delayed bulb initiation (Kinoshita et al., 2024). Conversely, late planting induced stress by reducing accumulated growing degree days, thereby limiting vegetative expansion and bulb size. These growth responses were effectively detected through differences in spectral reflectance, supporting the view that phenology-driven variability must be accounted for in yield prediction models (Kang et al., 2020). Sakamoto et al. (2013) similarly demonstrated that differential growth patterns across sowing windows can influence the accuracy of satellite-based biomass estimation. While NDVI remains a standard vegetation index in remote sensing because of its simplicity and widespread validation, its limitations under dense canopies necessitate the use of complementary indices. Indices such as GNDVI, which utilize green reflectance, are more effective in detecting subtle changes in chlorophyll content, particularly under moderate to high canopy densities (Delegido et al., 2011). These findings indicate that the selection of appropriate vegetation indices or index combinations should be tailored to the crop species and the specific growth stage targeted for prediction. Although this study employed UAVs at the field scale, scaling up to regional or national applications may require integration with satellite-based data. Nevertheless, the high spatial resolution of UAV imagery makes it valuable for calibrating and validating coarser-resolution remote sensing products. UAV platforms also provide flexible flight planning, enabling on-demand monitoring following weather events, irrigation, or early signs of crop stress. Integrating UAV-based vegetation indices with physiological models and crop simulation platforms such as DSSAT or APSIM represents an emerging area of interest. Such integration could facilitate the translation of spectral data into agronomic insights, including biomass partitioning, nutrient requirements, and harvest index estimation, thereby enabling real-time decision-making by farmers, researchers, and policymakers. The synergy between remote sensing, crop modeling, and Internet of Things (IoT) infrastructure represents a promising frontier for precision agriculture (Chlingaryan et al., 2018).

In the current study, random forest regression consistently performed well across both individual years and pooled datasets, demonstrating strong generalization. This was evidenced by low RMSE values (training: 1.911 t ha^−1^; cross-validation: 3.824 t ha^-1^) and MAE (training: 1.459 t ha^−1^; validation: 3.110 t ha^-1^), along with relatively high R^2^ values (training: 0.939; validation: 0.755), indicating the model’s ability to accurately explain yield variability at the bulb development stage. Comparable results were reported by Ramos et al. (2020), who predicted maize yield using UAV-based vegetation indices. Random forest has also been successfully applied to estimate the yield of bulbous vegetables such as garlic using UAV-based multispectral imagery across multiple sensors and phenological stages (Marcone et al., 2024). Random forest models are particularly effective at

Forest distinguishes itself by handling nonlinear relationships between predictor and target variables, making them well suited for complex agricultural modeling (Fernández-Delgado et al., 2014; Elbaşi et al., 2024). Moreover, they can capture complex interactions among environmental, soil, and biological factors (Meng et al., 2025; Sadasivam, 2021), which has led to their widespread adoption in yield prediction and other agricultural applications (Fu et al., 2025). Notably, the support vector machine with radial kernel also demonstrated strong generalization, with a smaller gap between training (R^2^ = 0.787) and validation (R^2^ = 0.716) performance compared with random forest, highlighting its robustness against overfitting. Support vector machines with radial kernels are well suited for handling complex, high-dimensional datasets with relatively limited sample sizes, a scenario common in field-based agricultural experiments (Fernández-Delgado et al., 2014; Kumar et al., 2025). Their kernel-based approach enables the capture of nonlinear patterns in canopy reflectance data while remaining less sensitive to multicollinearity than linear regression methods. These findings underscore the complementary strengths of random forest (high accuracy and flexible modeling) and svmRadial (robust generalization), supporting their reliability for yield prediction at specific crop growth stages in rainy-season onion.

This study validates the utility of UAV-derived multispectral imagery for predicting onion bulb yield across staggered planting dates. Vegetation indices showed significant associations with yield, particularly when measured during the bulb development stage. The effects of planting time, environmental conditions, and crop growth stage on vegetation index performance underscore the need for context-specific model calibration (Vidican et al., 2023). Integrating stage-specific spectral data with robust modeling approaches positions UAV-assisted remote sensing as a valuable tool for advancing onion agronomy. Ongoing advances in sensor technology, data analytics, and agronomic modeling will further improve the precision, scalability, and utility of these tools in supporting sustainable and profitable onion production systems (Din et al., 2021; Messina et al., 2020). The findings of this study are relevant not only for individual growers but also for researchers, policymakers, crop insurance agencies, and stakeholders throughout the supply chain. Early and accurate yield predictions can inform input allocation, price forecasting, and logistics management. For semi-perishable crops such as onion, where sudden gluts or shortages can lead to price volatility, remote sensing offers a proactive tool for large-scale crop area estimation and yield forecasting (Darwin et al., 2021; Pham et al., 2022). The study further underscores the importance of multi-year and multi-location validation of UAV-based yield prediction systems. Incorporating ground-truth measurements, such as chlorophyll meters, leaf area sensors, or plant biomass sampling, would enhance the calibration of predictive models. In addition, advances in cloud-based platforms, edge computing, and AI-driven analytics are making it increasingly feasible to analyze UAV data in near real time, reducing the lag between data acquisition and actionable insights (Samuel and Baysal-Gurel, 2019; Maes and Steppe, 2018). The findings of the present study offer practical applications for precision crop management, yield forecasting, and advisory services by enabling accurate, field-scale prediction of onion yield using UAV-based multispectral data. However, high drone costs, shortages of trained professionals, and data acquisition complexities—particularly when integrating UAV data with satellite imagery—as well as privacy concerns may limit widespread adoption. Additionally, UAVs face constraints related to large-area monitoring and weather dependency, whereas satellite imagery, although broader in spatial coverage, provides lower spatial resolution, directly affecting the scale and precision of agricultural monitoring and yield prediction (Bazrafkan et al., 2025; Toosi et al., 2025). For scalable adoption, future research should prioritize automated UAV workflows, capacity building for technical operators, and the development of hybrid UAV–satellite systems, along with socioeconomic evaluations to support broad and sustainable implementation across diverse farming systems (Jabed and Azmi Murad, 2024).

## 5 Conclusion

This study demonstrated the potential of UAV-based multispectral imagery for effective yield prediction in rainy-season onion, evaluated across four planting dates over two consecutive years (2023 and 2024) in India. A more significant correlation between vegetation indices and bulb yield was observed in 2024 compared to 2023, underscoring interannual variability. Among the growth stages evaluated, the bulb development stage consistently showed the strongest association with yield in both years. To further address interannual variability, future research should incorporate a wider range of planting dates, multiple seasons and years, and diverse agro-climatic zones. Among the spectral indices tested, NDVI, NDRE, GNDVI, and NORM2 demonstrated the strongest correlations with bulb yield across both years. Of the five machine learning algorithms employed, random forest (rf) and support vector machine with radial basis kernel (svmRadial) models effectively captured yield variability and generalized well for prediction. Overall, the findings confirm that UAV-acquired multispectral imagery, combined with robust modeling approaches, offers a reliable and scalable solution for predicting onion yield under rainy-season cultivation.

## Data Availability

The raw data supporting the conclusions of this article will be made available by the authors, without undue reservation.
