# Supplementary material for: UAV multispectral sensing and data-driven modeling for precision onion yield prediction
Source: Front Plant Sci. 2026 Feb 6;16:1696730. doi: 10.3389/fpls.2025.1696730 (PMC12921482; doi:10.3389/fpls.2025.1696730)
Supplement: Supplementary file 1 [file DataSheet1.docx]

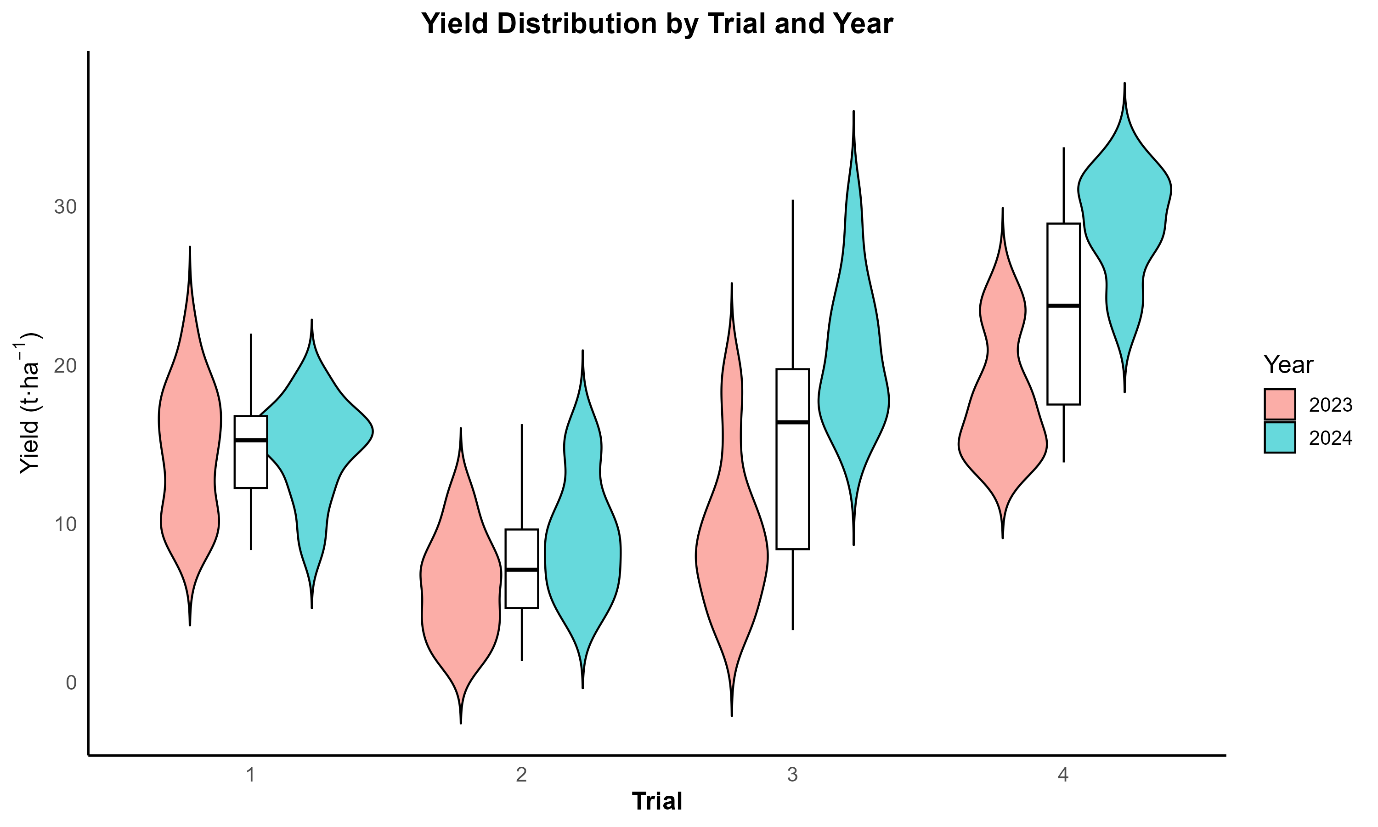


Figure S1. Violine plots showing the distribution of onion yield (t.ha^-1^) across different trials and years (2023 and 2024). Each violine represent the yield variability within trial, with the boxplot embedded inside indicating the interquartile range, median, and extremes.


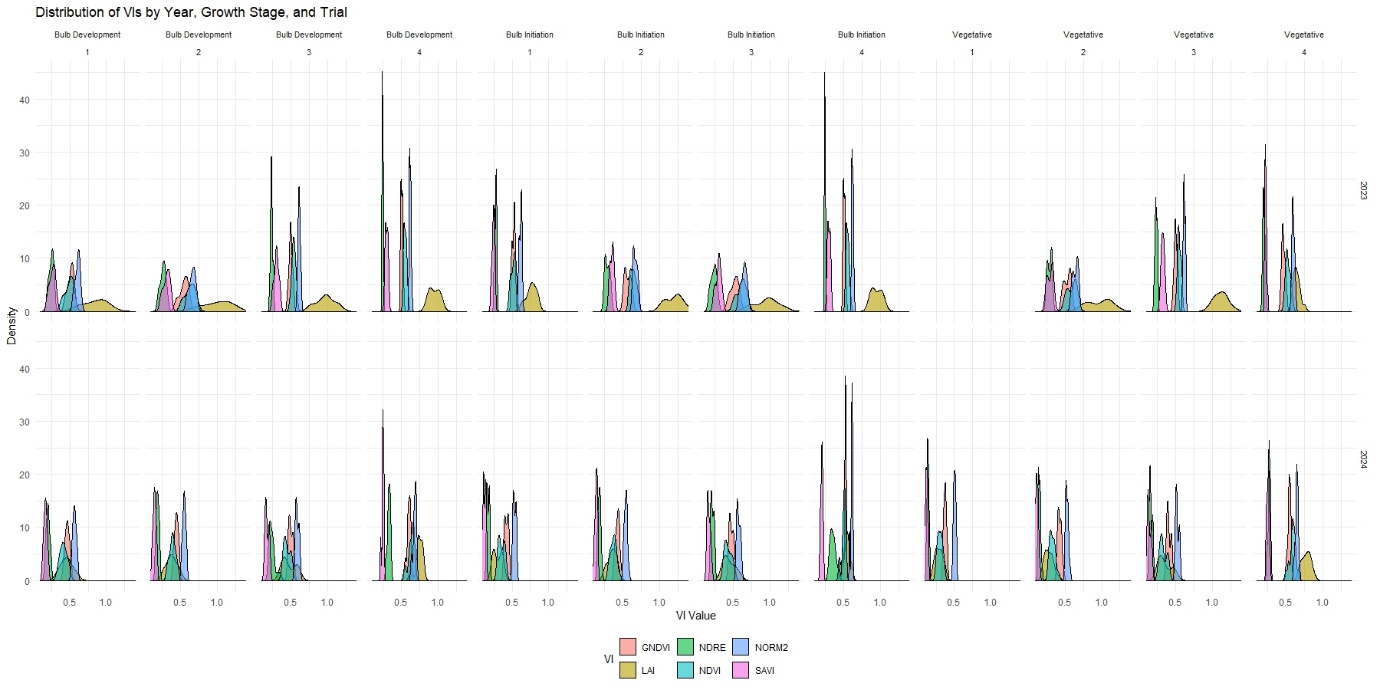


Figure S2. Density distribution plot of vegetative indices (VIs) across three crop growth stages (Vegetative, bulb initiation and bulb development) for both year (2023 and 2024).


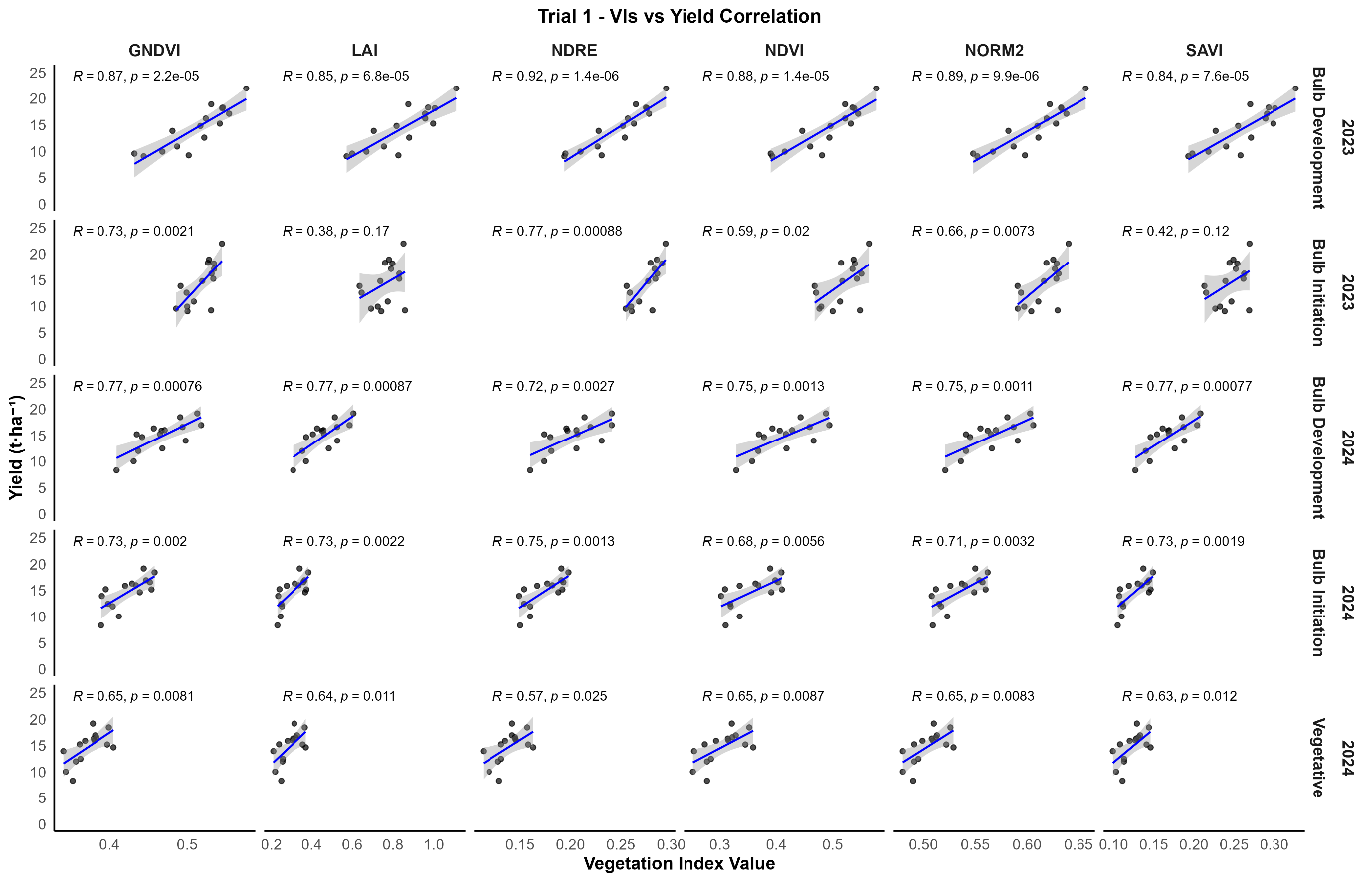


Figure S3. Pearson’s correlation between vegetative indices and onion yield at different growth stages in Trial 1 (2023 and 2024).


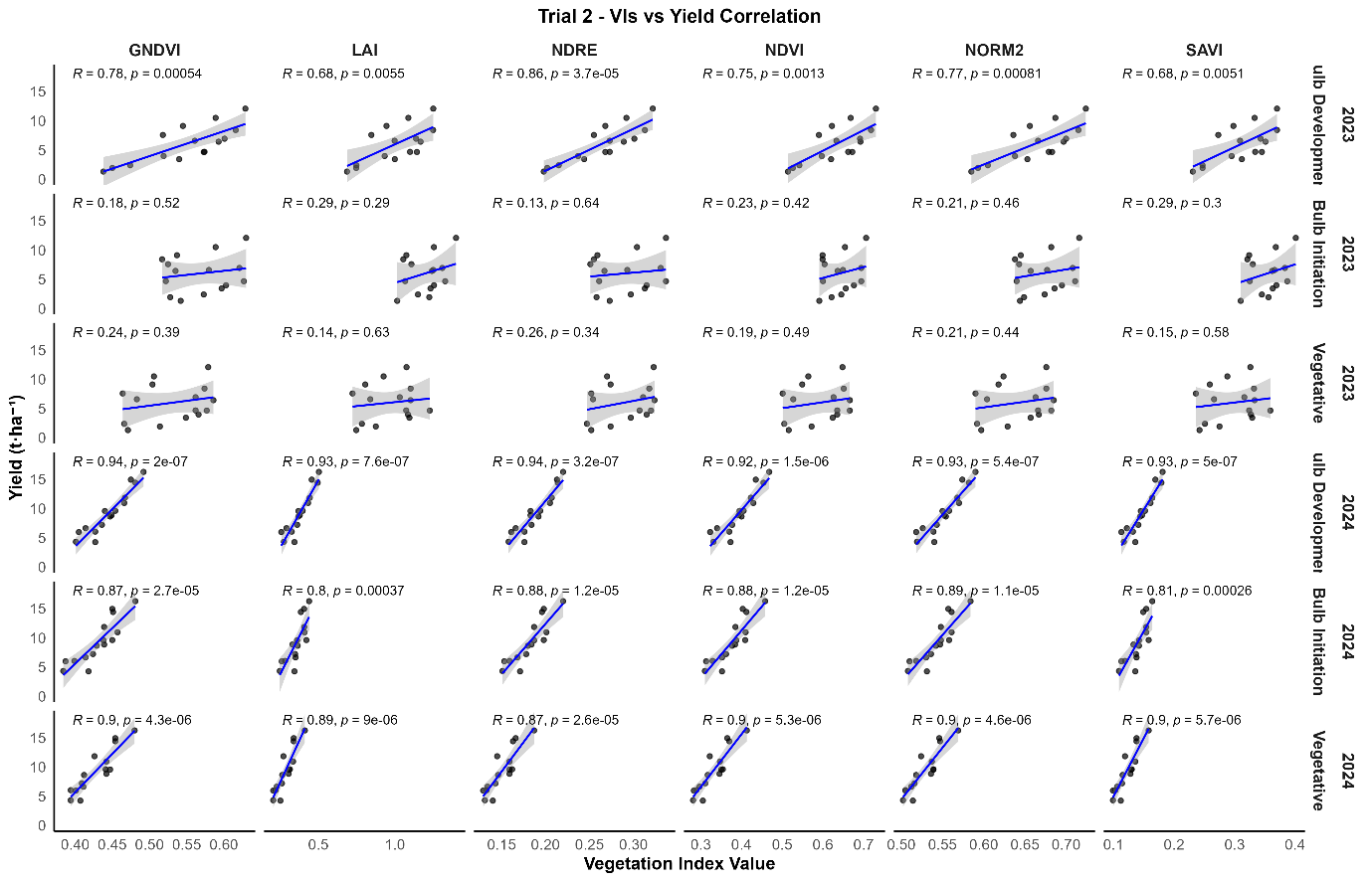


Figure S4. Pearson’s correlation between vegetative indices and onion yield at different growth stages in Trial 2 (2023 and 2024).


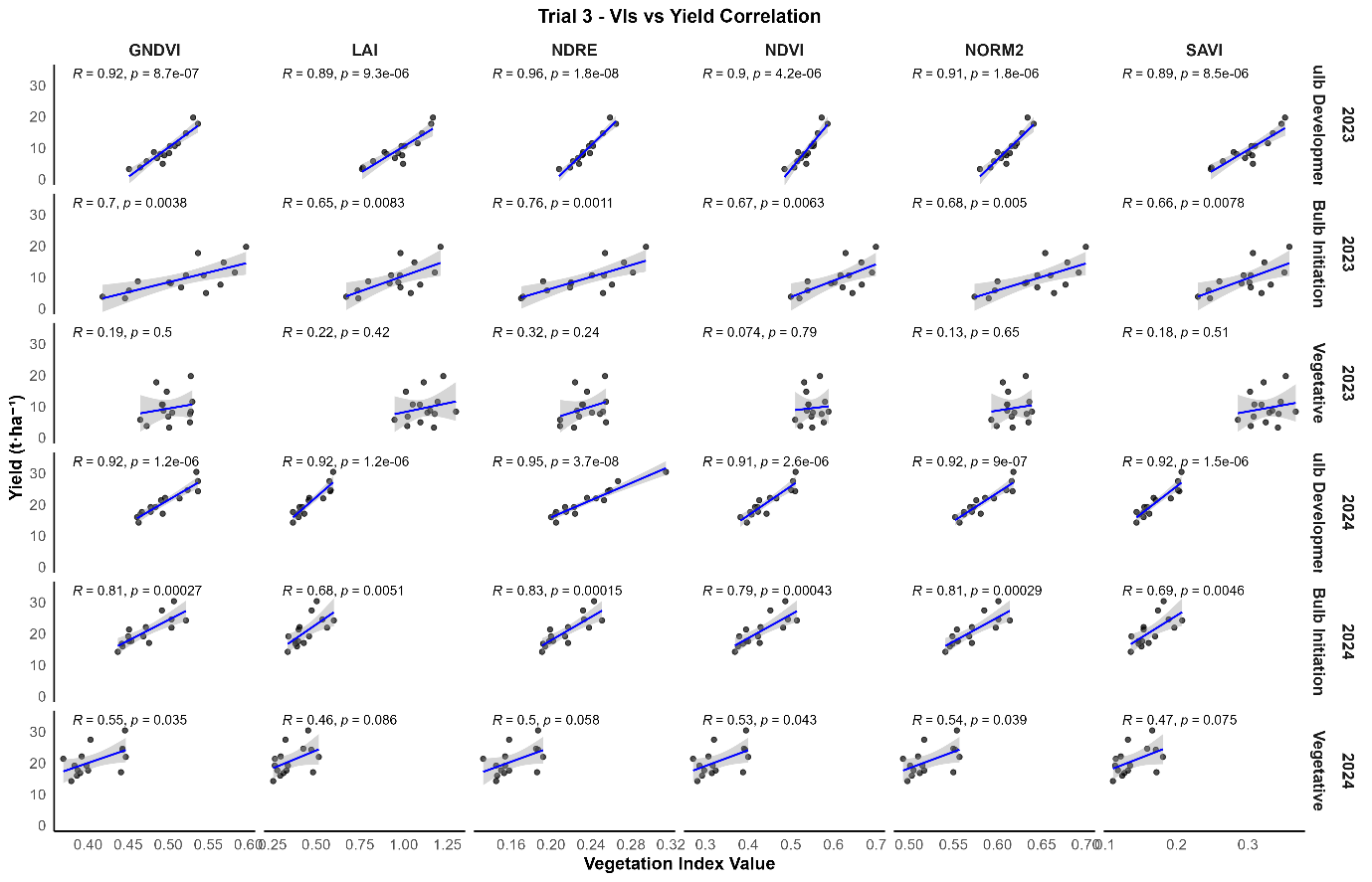


Figure S5. Pearson’s correlation between vegetative indices and onion yield at different growth stages in Trial 3 (2023 and 2024).


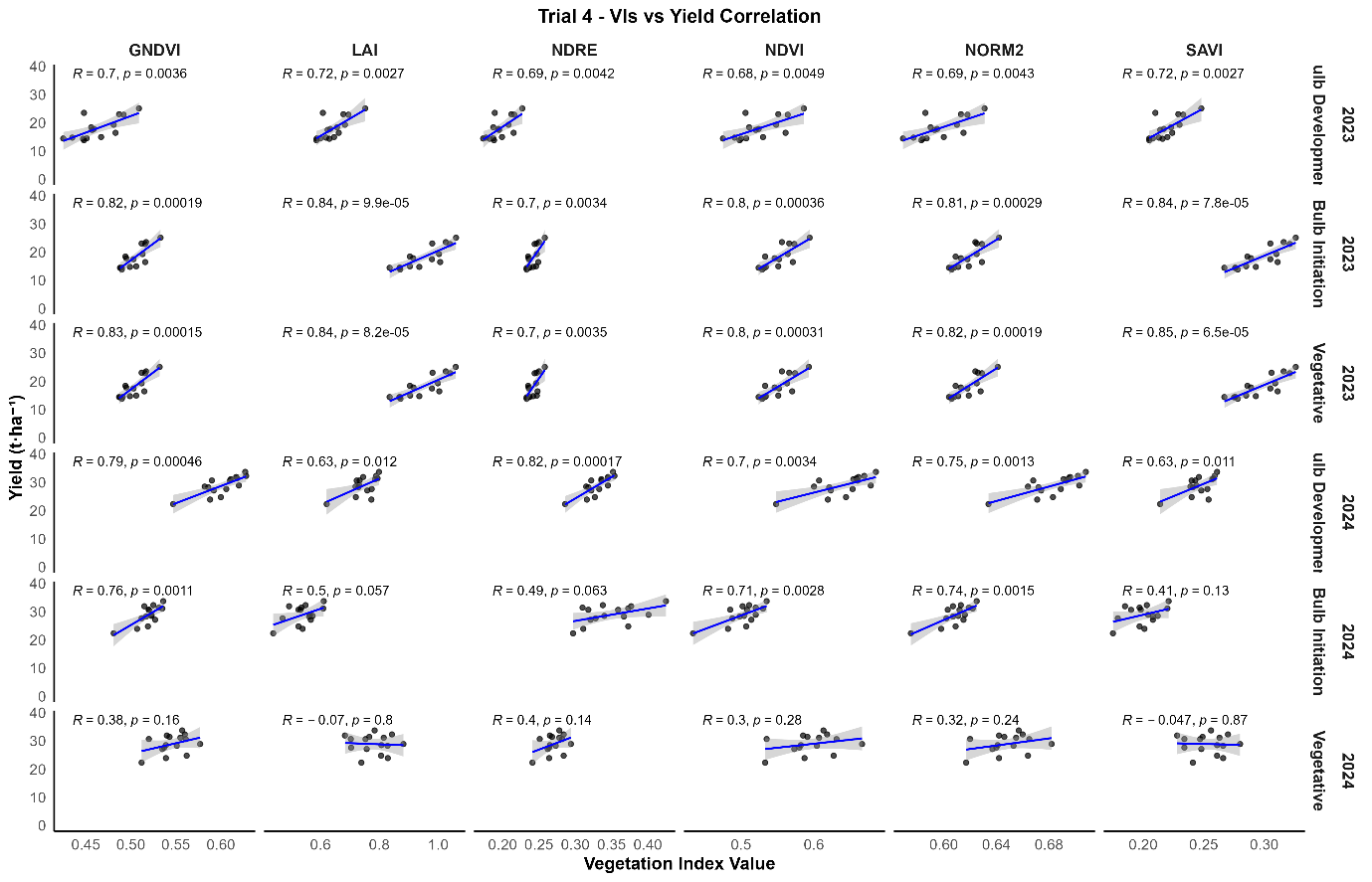


Figure S6. Pearson’s correlation between vegetative indices and onion yield at different growth stages in Trial 4 (2023 and 2024).


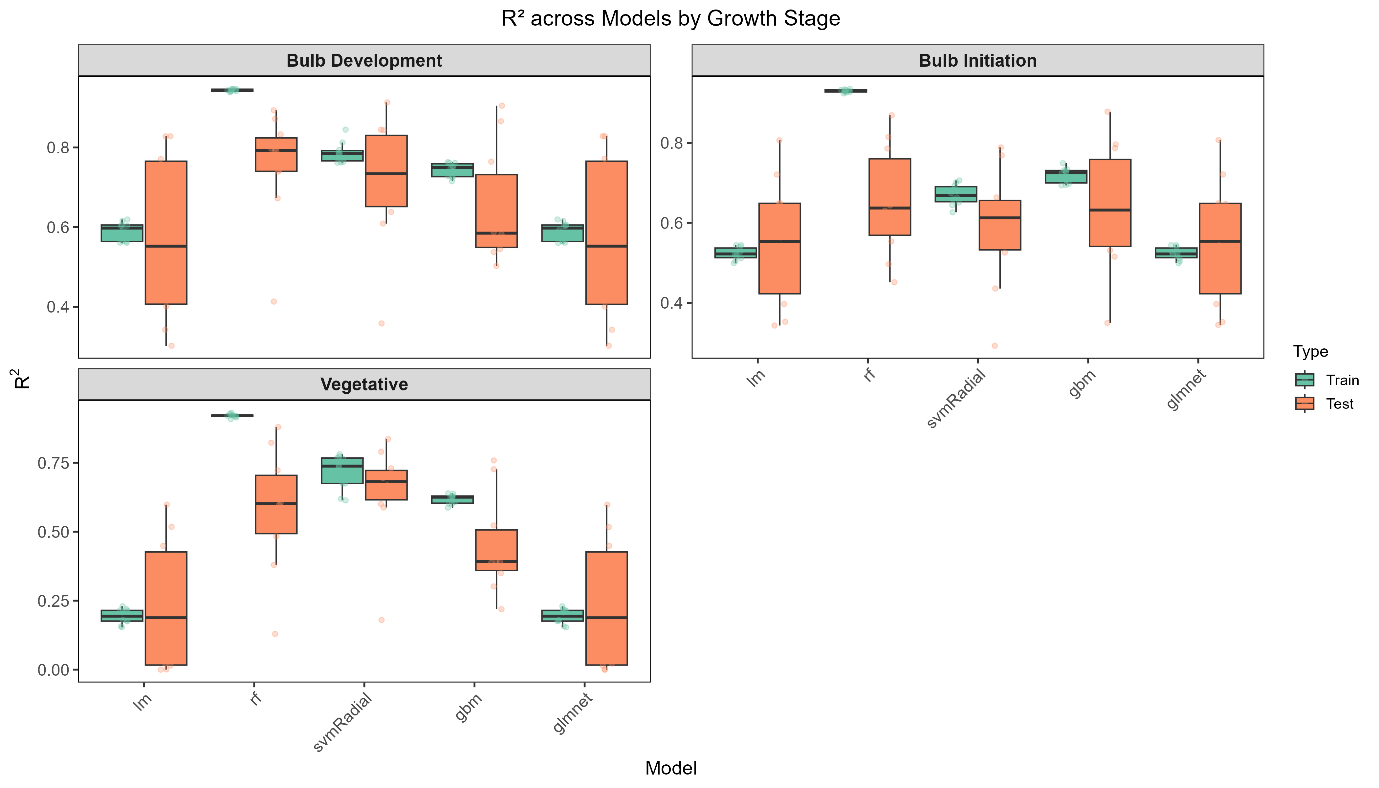


Figure S7. Boxplot of model R^2^ value for training and cross-validation dataset across ML algorithms using 10-fold cross-validation on combined data (2023-2024).


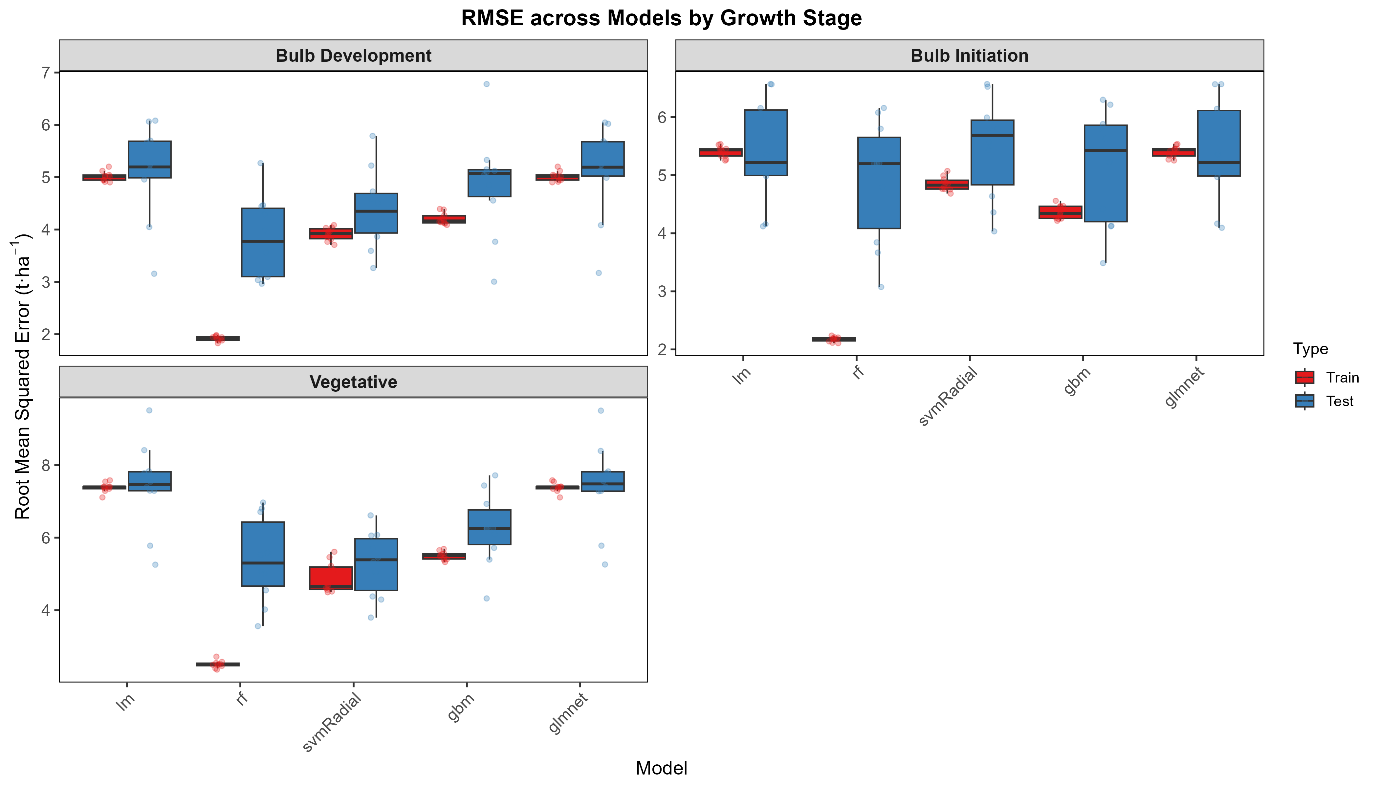


Figure S8. Boxplot of model RMSE value for training and cross-validation dataset across ML algorithms using 10-fold cross-validation on combined data (2023-2024).


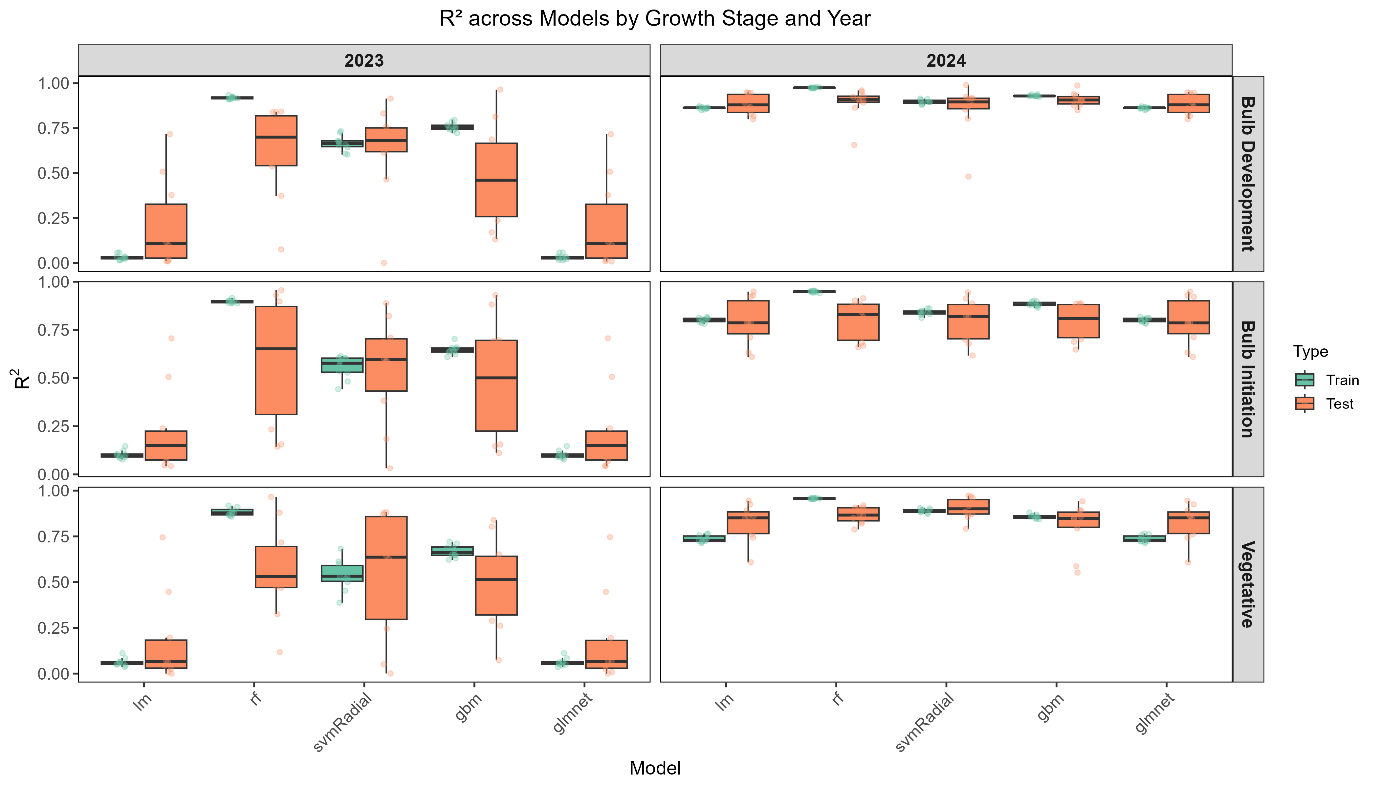


Figure S9. Boxplot of model R^2^ value for training and cross-validation dataset across ML algorithms using 10-fold cross-validation on 2023 data and 2024 data.


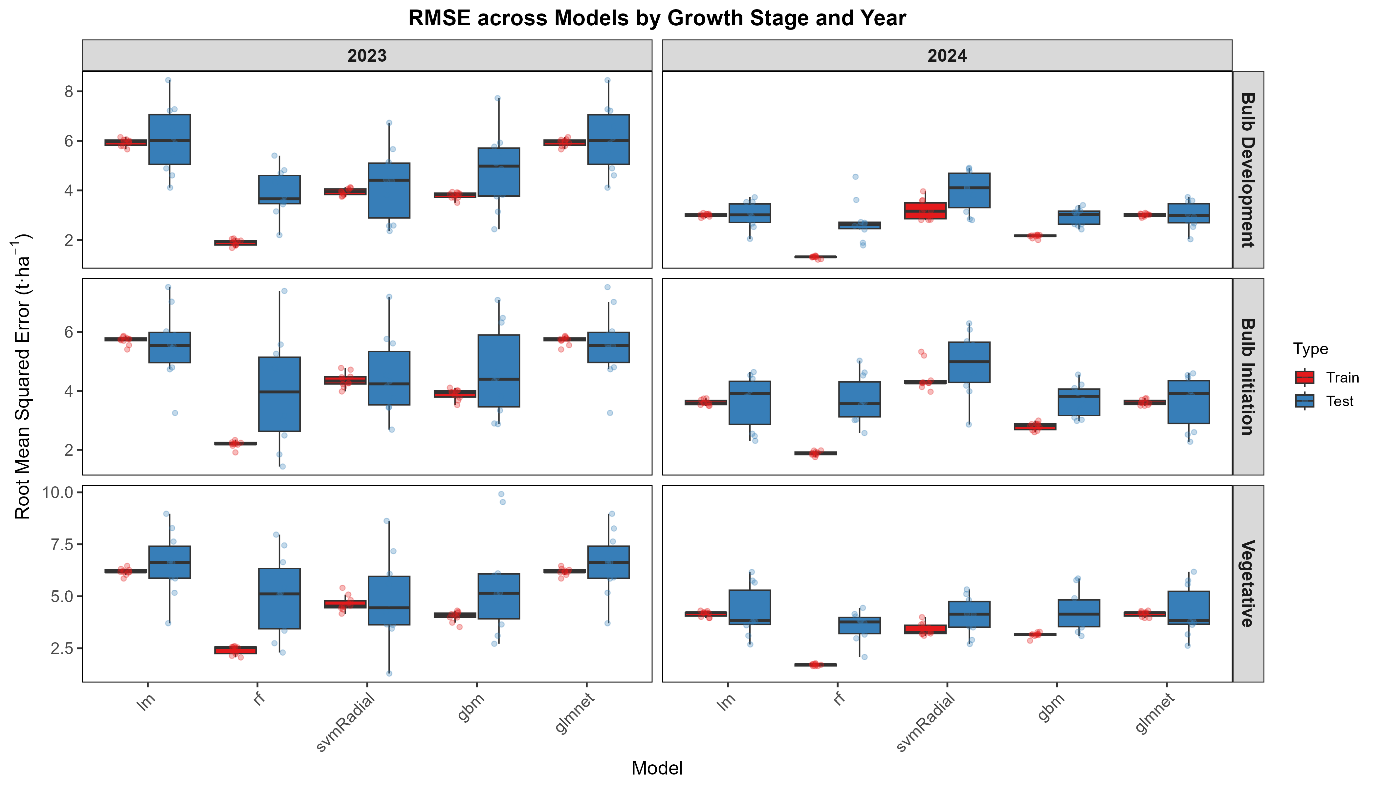


Figure S10. Boxplot of model RMSE value for training and cross-validation dataset across ML algorithms using 10-fold cross-validation on 2023 data and 2024 data.

The plot S7-S10 illustrates the distribution of R^2^ and RMSE values achieved by five ML models – gbm, glmnet, ln, rf, and svmRadial at different growth stages. Consistent performance across fold in both training and validation sets demonstrates models’ stability and generalization capacity for rainy season onion yield prediction.
